# Autophagy inhibits cancer stemness in triple‐negative breast cancer via miR‐181a‐mediated regulation of ATG5 and/or ATG2B

**DOI:** 10.1002/1878-0261.13180

**Published:** 2022-01-26

**Authors:** Jee Won Park, Yesol Kim, Soo‐been Lee, Chae Won Oh, Eun Ji Lee, Je Yeong Ko, Jong Hoon Park

**Affiliations:** ^1^ Department of Biological Science Sookmyung Women’s University Seoul Republic of Korea

**Keywords:** ATG2B, ATG5, autophagy, cancer stemness, miR‐181a, triple‐negative breast cancer

## Abstract

Autophagy has a dual role in the maintenance of cancer stem cells (CSCs), but the precise relationship between autophagy and cancer stemness requires further investigation. In this study, it was found that luminal and triple‐negative breast cancers require distinct therapeutic approaches because of their different amounts of autophagy flux. We identified that autophagy flux was inhibited in triple‐negative breast cancer (TNBC) CSCs. Moreover, miRNA‐181a (miR‐181a) expression is upregulated in both TNBC CSCs and patient tissues. Autophagy‐related 5 (ATG5) and autophagy‐related 2B (ATG2B) participate in the early formation of autophagosomes and were revealed as targets of miR‐181a. Inhibition of miR‐181a expression led to attenuation of TNBC stemness and an increase in autophagy flux. Furthermore, treatment with curcumin led to attenuation of cancer stemness in TNBC CSCs; the expression of ATG5 and ATG2B was enhanced and there was an increase of autophagy flux. These results indicated that ATG5 and ATG2B are involved in the suppression of cancer stemness in TNBC. In summary, autophagy inhibits cancer stemness through the miR‐181a‐regulated mechanism in TNBC. Promoting tumor‐suppressive autophagy using curcumin may be a potential method for the treatment of TNBC.

## Graphical abstract

Triple‐negative breast cancer (TNBC) is the most aggressive subtype of breast cancer. However, potential therapeutic targets in TNBC have not been fully elucidated. In this study, we identified the tumor‐suppressive role of miR‐181a and investigated the role of miR‐181a in regulating autophagy and TNBC stemness. Inhibition of miR‐181a elicited tumor‐suppressive effects by inducing autophagy and may thus be considered as a potential therapeutic strategy in patients with TNBC.

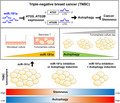

**Abbreviations**
3′ UTR: 3′ Untranslated region
ALDH: aldehyde dehydrogenase
ATG2B: autophagy‐related 2B
ATG5: autophagy‐related 5
CCK‐8: cell counting kit‐8
CD24: cluster of differentiation 24
CD44: cluster of differentiation 44
CQ: chloroquine
CRISPR: clustered regularly interspaced short palindromic repeats
CSC: cancer stem cell
DAPI: 4',6‐Diamidino‐2‐Phenylindole
DMEM: Dulbecco's modified Eagle medium
DMSO: dimethyl sulfoxide
EBSS: earle's balanced salt solution
ER: estrogen receptor
HADb: human autophagy database
HER2: human epidermal growth factor receptor 2
ICC: immunocytochemistry
KO: knockout
LAMP2: lysosome‐associated membrane glycoprotein 2
LC3: microtubule‐associated proteins 1A/1B light chain 3B
miRNA: microRNA
MRE: miRNA regulatory elements
NANOG: homeobox protein NANOG
NSCLC: non‐small‐cell lung carcinoma
OCT4: octamer‐binding transcription factor 4
p62: ubiquitin‐binding protein p62
PR: progesterone receptor
qPCR: quantitative polymerase chain reaction
qRT‐PCR: quantitative reverse transcription PCR
SOX2: SRY (sex determining region Y)‐box 2
TNBC: triple‐negative breast cancer

## 1 Introduction

Breast cancer is one of most common cancers in women. It is usually classified into four subtypes, depending on the type of receptor expressed. Triple‐negative breast cancer (TNBC) is one of the four subtypes that lack the expression of estrogen receptor (ER), progesterone receptor (PR), and human epithermal growth factor receptor 2 (HER2) [1]. Among breast cancer patients, 15–20% of women are diagnosed as triple‐negative [2]. TNBC is known to be the most aggressive type of all breast cancer subtypes and has the worst prognosis of the four subtypes. Since TNBC cells do not express endocrine receptors on their surface, therapies such as anti‐cancer drugs, which target receptors on the cancer cells, cannot be used. As a result, therapies for TNBC patients are limited to surgery, radiation therapy, and chemotherapy. For these reasons, there is an imminent need to identify new targets for TNBC therapy. An increasing number of studies have identified miRNAs as potential biomarkers for human cancer diagnosis, prognosis, and therapeutic targets/tools [3].

Cancer stem cells (CSCs) are a certain type of cancer cells that display stem cell‐like properties, that is, the pluripotent capacity to differentiate into all cell types. Recently, it has been found that CSCs are immortal tumor‐initiating cells that comprise a small subset of all cancer cells. CSCs have self‐renewal abilities and have been reported to cause cancer genesis, growth of tumor, recurrence, and drug resistance [4]. Complete elimination of the CSC population, in order to prevent tumor recurrence and to provide complete treatment, has been suggested as an important approach in cancer therapy. Therefore, CSCs emerge as an important potential target for therapeutic intervention. Therapeutic strategies that use CSCs as targets have been suggested as a way to increase the efficiency of conventional anticancer therapies [5]. Previous studies have demonstrated that TNBC displays more CSC‐like characteristics than other breast cancer subtypes [6]. Upon such examination, it has been found that the incidence of the CD44^+^/CD24^‐^ population is significantly higher in TNBC than in other breast cancer subtypes [7, 8]. Previous studies have identified that CSC enrichment frequently arises in response to chemotherapy. The relative resistance of CSCs to chemotherapy, particularly in case of TNBC, has been speculated to be a key mechanism for relapse [9, 10]. These previous studies suggest CSCs as one of the factors responsible for the aggressiveness of TNBC.

Macroautophagy (referred to as autophagy) is a molecular mechanism by which cellular waste or damaged organelles are degraded. Autophagy has been reported to be related to the occurrence and survival of cancer cells. A number of previous studies have reported the role of autophagy as a tumor suppressor [11]. Autophagy plays a critical role in cytoprotection and maintaining homeostasis by preventing the accumulation of toxic proteins and recycling cytoplasm to generate energy under stress conditions [12]. Previous studies have reported that the effect of autophagy on the survival of breast cancer cells depends on the subtypes [13, 14]. Due to differences in the role of autophagy, each subtype requires a different therapeutic application of autophagy. Additionally, recent studies indicate that autophagy plays a key role in the maintenance of CSCs [15].

This study aims to explore the role of autophagy on the regulation of cancer stemness in TNBC and the mechanism of regulation, in which miR‐181a is involved. In the present study, we aimed to investigate whether downregulation of the autophagic molecules by miR‐181a effects the maintenance of cancer stemness in TNBC.

## 2 Materials and methods

### 2.1 Cell culture

MDA‐MB‐231, MDA‐MB‐231/A, MCF7 cells, and HEK293T cells were grown in Dulbecco’s modified Eagle’s medium (DMEM; WelGENE Inc., Gyeongsan, South Korea) with 10% (FBS; Gibco; Thermo Fisher Scientific Inc., Waltham, MA, USA) and 1% penicillin/streptomycin (WelGENE Inc.) at 37 °C, 5% CO_2_ atmosphere. MDA‐MB‐231, MCF7 cells were provided by Sapporo Medical University. The MDA‐MB‐231/A cells and primary mouse tumor cells that have been used in this study were provided by Yonsei University [16]. All the cell lines were subjected to DNA fingerprinting analysis (Korean Cell line Bank) in 2020.

### 2.2 Induction and inhibition of autophagy

In order to establish the starved condition and induce autophagy, cells were incubated in Earle’s balanced salt solution (EBSS; WelGENE Inc.) for 4 h. For treatment of curcumin, cells were plated at a density of 1 × 10^5^ cells·mL^−1^. 24 h after cell plating, cells were treated with curcumin (Sigma‐Aldrich, Saint Louis, MO, USA) diluted in dimethyl sulfoxide (DMSO; Sigma‐Aldrich) for 6 h. MDA‐MB‐231/A cells were treated with 50 μm curcumin, and MDA‐MB‐231/A tumorspheres were treated with 25 μm curcumin. MDA‐MB‐231 cells were treated with 30 μm curcumin. Rapamycin (Sigma‐Aldrich) was used to induce autophagy and treated at 100–500 nm concentration for 24 h. To inhibit the autophagosome–lysosome fusion [17], chloroquine (CQ; Sigma‐Aldrich) was diluted in DMSO, and the cells were treated with 10 μm chloroquine for 3 h.

### 2.3 Western blotting analysis

Protein extracts were prepared using Nucleospin® DNA/RNA/protein kit (Macherey‐Nagel, Dueren, Germany). The concentration of protein was measured using a bicinchonic acid solution (Sigma‐Aldrich) and copper (Ⅱ) sulfate solution (Sigma‐Aldrich), using multi‐well spectrophotometer (Synergy HTX, BioTek Instruments, Winooski, VT, USA). Proteins (10–70 μg) were loaded onto 6–12% SDS/PAGE gel, electrophorased, and transferred onto polyvinylidene difluoride membranes (ATTO, Tokyo, Japan). The following primary antibodies were used in this study: ATG5 (1 : 2000; Cell Signaling Technology, USA), ATG16L1 (1 : 2000; Cell Signaling Technology, Danvers, MA, USA), ATG7 (1 : 2000; Cell Signaling Technology), ATG2B (1 : 800; Abcam, Cambridge, UK), ULK1 (1 : 2000; Cell Signaling Technology), LAMP2A (1 : 2000; Abcam), UVRAG (1 : 2000; Abcam), LC3A/B (1 : 2000; Cell Signaling Technology), p62 (1 : 8000; Novus Biologicals, Littleton, CO, USA, Santa Cruz Biotechnology, Dallas, TX, USA), OCT4 (1 : 2000; Abcam), SOX2 (1 : 2000, Abcam), and β‐actin (1 : 8000; Bethyl Laboratories, Montgomery, TX, USA). β‐actin was used as loading controls. Immunoreactive proteins were detected using horseradish peroxidase‐conjugated secondary antibodies (1 : 4000; Enzo Life Sciences, Farmingdale, NY, USA) and the enhanced chemiluminescence reagent, EzWestLumi plus (ATTO). All antibodies were diluted with skimmed milk (BD, Franklin Lakes, NJ, USA) in phosphate‐buffered saline (PBS; WelGENE Inc.) containing Tween® 20 (Sigma‐Aldrich).

### 2.4 Immunofluorescence staining

The tissues processed on a tissue array slide (BC081120f, US Biomax Inc, US Biomax Inc, Rockville, MD, USA) were deparaffinized and rehydrated in HistoClear and serially diluted ethanol (Merck, Darmstadt, Germany). After heat‐induced antigen retrieval, tissues were blocked with normal horse serum for 1 h and incubated overnight at 4 °C with primary antibodies. OCT4 (1 : 400; Abcam) and p62 (1 : 200; Santa Cruz Biotechnology) antibodies were used. Next, nuclei were counterstained with 4′,6‐diamidino‐2‐phenylindole (DAPI, Sigma‐Aldrich) for 15 min and 2 h at room temperature with Alexa Flour® 488‐ and 594‐conjugated secondary antibodies (1 : 1000; Enzo Life Sciences). The slide was mounted with a cover slide using mounting solution (Dako; Agilent Technologies, Santa Clara, CA, USA). Images were obtained with confocal laser scanning microscopy (LSM 700, Zeiss, Oberkochen, Germany) using identical exposure settings. The number of puncta was measured using i‐Solution software (version 22.5, InnerView™, Seongnam, South Korea). The intensity of nuclear OCT4 fluorescence was measured using imagej [18].

### 2.5 Preparation of breast cancer patient tissues

To observe the expression of cancer stemness and autophagy in human tissues, human breast cancer samples were surgically resected from patients. The study was conducted in accordance with the Declaration of Helsinki and was approved by the Institutional Review Board of Seoul National University Hospital (IRB number: 1704‐019‐843). Informed consent was obtained from all patients before collection of the specimens. Also, breast cancer tissue array (BC081120f, US Biomax Inc) was also purchased for immunofluorescence staining.

### 2.6 Transfection

To regulate miRNA expression, cells were plated at a density of 9 × 10^4^ cells·mL^−1^ and transiently transfected with 30nM of mirVana™ miR‐181a‐5p inhibitor (Ambion; Thermo Fisher Scientific Inc., Waltham, MA, USA) or mirVana™ negative control inhibitor (Negative Control #1, Ambion) using siPORT™ NeoFX™ transfection agent (Invitrogen; Thermo Fisher Scientific Inc., Waltham, MA, USA). For plasmid transfection, FuGene (Promega, USA) transfection reagent was used. All transfections were conducted for 24–72 h, according to the manufacturer’s instructions.

### 2.7 mRNA extraction, reverse transcription, and quantitative real‐time PCR

mRNA was extracted using the Nucleospin® DNA/RNA/protein kit (Macherey‐Nagel). RT‐PCR and quantitative real‐time PCR (qPCR) for quantification of mRNA expression were performed using oligo dT (Bioneer, Daejeon, South Korea), M‐MLV reverse transcriptase (Promega, Madison, WI, USA), and set of dNTP (Promega).

Following reverse transcription, 100 ng of each individually synthesized cDNA was used in the qPCR with SYBR Green qPCR master mix (PCR Biosystems, London, UK) and primers designed for each OCT4, SOX2, NANOG, ATG5, ATG2B (Bionics, Seoul, South Korea). Human 18s ribosomal RNA was used as the endogenous control. The sequences of qPCR primers are described in Table S1. All quantitative PCR (qRT‐PCR) analyses were performed using LightCycler® 96 system (Roche, Basel, Switzerland), as described previously [19].

### 2.8 Total RNA isolation, miRNA RT‐PCR, and qRT‐PCR

Total RNA was isolated using TRIzol® reagent (Ambion) and the clean‐up was performed using chloroform (Sigma‐Aldrich) and isopropanol (Merck). For quality control, RNA purity and integrity were evaluated by measurement of the OD 260/280 ratio. RT‐PCR was performed using 100–500 ng of total RNA. TaqMan® microRNA assays and TaqMan® microRNA reverse transcription kit (Applied Biosystems; Thermo Fisher Scientific Inc., Waltham, MA, USA) were used for RT‐PCR. TaqMan® probes for RT‐PCR were used for reverse transcription of miR‐181a (miRBase ID: miR‐181a‐5p, Assay ID: 000480, Applied Biosystems). qPCR for miRNA analysis was performed using TaqMan® universal master mix (Applied Biosystems). A small nuclear RNA, C/D Box 48 (*RNU48*) RNA, was used as the endogenous control (Assay ID: 001006, Applied Biosystems).

### 2.9 Immunocytochemistry

Immunocytochemistry (ICC) of the tumorspheres was performed referring to a previously published protocol [20]. Briefly, cells were seeded onto poly‐l‐lysine‐coated coverslips and grown in stem cell medium for 3–10 days. After the tumorspheres were formed, cells were fixed in 10% neutral‐buffered formalin (Biosesang, Seongnam, South Korea) for 30 min. Following fixing, the tumorspheres were permeabilized for 5 min and then blocked using 1% BSA (Bovogen, Keilor East, Australia) in PBS (WelGENE Inc.) for 30 min. The cells were then incubated with primary antibodies for 1 h in an incubator maintained at 37 °C. The following primary antibodies were used in this study: OCT4 (1 : 400; Abcam), p62 (1 : 200; Santa Cruz Biotechnology), LC3B (1 : 200; Cell Signaling Technology), and LAMP2 (1 : 200; Santa Cruz Biotechnology). Next, the cells were incubated with Alexa Flour® 488‐ and 594‐conjugated secondary antibodies (1 : 1000; Enzo Life Sciences) for 1 h at room temperature. Lastly, the cells were stained with DAPI‐containing mounting solution (Dako). The cells were visualized using a confocal laser scanning Microscope (LSM 700, Zeiss), and all the images were captured using identical exposure settings. The number of puncta was measured using i‐Solution software (version 22.5, InnerView™). The intensity of nuclear OCT4 fluorescence was measured using imagej [18].

### 2.10 Tumorsphere formation assay

MDA‐MB‐231, MDA‐MB‐231/A, and MCF7 cells were plated in low‐attachment culture dishes at a density of 10  000–20 000 cells·mL^−1^ in DMEM/F12 basic medium, supplemented with 2% B27 (Gibco), 20 ng·mL^−1^ human epidermal growth factor (Prospec, Rehovot, Israel), 20 ng·mL^−1^ basic human fibroblast growth factor (Gibco), 4 μg·mL^−1^ heparin (Sigma‐Aldrich), and 30% bovine serum albumin (BSA; Sigma‐Aldrich). Cells were grown for 3–10 days in suspension at 37 °C in a 5% CO_2_ atmosphere. Tumorsphere volume area was measured using imagej [18]. The numbers of tumorspheres were counted under a microscope [21].

### 2.11 Dual‐luciferase reporter assay

The 3′ UTRs of human ATG5 and ATG2B, which included the predicted miR‐181a seed sequence, were PCR‐amplified using HEK293T complementary DNA as the template. The primers used for amplification of 3’UTRs are listed in Table S2. The amplified 3′ UTRs were cloned into pmirGLO Dual‐Luciferase® miRNA Target Expression vector (Promega) using the In‐fusion® HC Cloning kit (Clontech Laboratories, Mountain View, CA, USA). The luciferase constructs were cotransfected along with mirVana™ miR‐181a‐5p mimic (Ambion) or mirVana™ negative control mimic (Negative Control #1, Ambion) into HEK293T cells, using Lipofectamine 2000 (Invitrogen). Cells were lysed after 24–48 h, and the luciferase activities were measured using multiwell spectrophotometer (Synergy HTX, BioTek Instruments, USA) and Dual‐Luciferase® Reporter Assay System (Promega), according to the manufacturer’s instructions. The relative luciferase activities were normalized to the *Renilla* luciferase activity of the pmirGLO vector.

### 2.12 Target prediction database analysis

To obtain the list of miR‐181a predictive target genes involved in the autophagy pathway, two target prediction databases were analyzed—TargetScan [22] and Human Autophagy Database (HADb, http://www.autophagy.lu/).

### 2.13 Acridine orange staining

Acridine orange staining was performed to detect the formation of autolysosomes in MDA‐MB‐231/A cells. Cells were incubated for 15 min in complete medium containing 1 μg·mL^−1^ Acridine orange hemi (zinc chloride) salt (Sigma‐Aldrich). Cells were then stained for 5 min with Hoechst 33342 (Invitrogen) diluted 1 : 2000 in PBS, according to the manufacturer’s instructions. After the staining solution was removed, the cells were washed thrice with PBS and imaged using confocal laser scanning microscopy (LSM 700, Zeiss).

### 2.14 Aldefluor Assay

To identify the percentage of CSCs with high ALDH activity in MDA‐MB‐231/A cell population, ALDEFLUOR^TM^ kit (Stemcell Technologies, Vancouver, BC, Canada) was used, according to the manufacturer’s instructions and methods of previous studies [23, 24]. MDA‐MB‐231/A cells were grown on coverslips. After treatment of miR‐181a inhibitor for 48 h and curcumin for 6 h, coverslips were washed with Hank’s buffered salt solution (HBSS; WelGENE Inc.). Cells were then stained with activated Aldefluor reagent with nuclear stain Hoechst 33342 and incubated for 45 min at 37°C. The coverslips were washed with HBSS and imaged using confocal laser scanning microscopy. The percentage of MDA‐MB‐231/A cells containing ALDH puncta was measured only on cells that were not DEAB treated.

### 2.15 CCK‐8 Assay

Cell proliferation was measured using a cell counting kit‐8 (CCK‐8; Dojindo Laboratories, Kumamoto, Japan), according to the manufacturer’s instructions. MDA‐MB‐231/A stable miR‐181a KO cells were plated in 24‐well plates at a density of 1 × 10^4^ cells·mL^−1^. Cells were first plated in DMEM with 10% FBS. After 4 h, the culture medium was replaced with DMEM with 1% FBS. Then, CCK‐8 reagent was added to a single well of each group and incubated for 30 min at 37°C. The absorbance at 450 nm was measured using a multiwell spectrophotometer (Synergy HTX, BioTek Instruments). Cell proliferation was measured at 48 h intervals for 4 days.

### 2.16 Cyto‐ID^®^ autophagy detection dye staining

Cyto‐ID® autophagy detection kit (Enzo Life Sciences), which includes novel dye that selectively labels accumulated autophagic vacuoles, was used to monitor autophagy flux in live cells. MDA‐MB‐231/A cells were plated on coverslips at a density of 9 × 10^4^ cells·mL^−1^ and treated with miRNA inhibitor or curcumin. Post‐treatment, the medium was removed and cells were stained with Cyto‐ID® autophagy detection dye (Enzo Life Sciences) according to the manufacturer’s instructions. Nuclei were conterstained with Hoechst 33342, according to the manufacturer’s instructions. Cells were imaged using confocal laser scanning microscopy.

### 2.17 Limiting dilution assay

MDA‐MB‐231/A cells in the 200 µL of tumorsphere assay medium were seeded into 96‐well plates for culturing tumorspheres. The number of wells containing tumorspheres was counted using a light microscope 2–3 weeks after plating. Cancer cell‐initiating frequency and significance were analyzed using the online software ELDA: extreme limiting dilution analysis [25].

### 2.18 Construction of CRISPR‐knockout (KO) cell line

The cloning process was performed according to the manufacturer’s protocols [26]. The lentiCRISPR v2 vector (Addgene, Watertown, MA, USA) was digested using BsmBI (NEB, Ipswich, MA, USA). To knockout the chromosome site of miR‐181a‐1 and miR‐181a‐2, two pairs of oligos were designed using an online tool named CHOPCHOP (http://chopchop.cbu.uib.no/) and cloned into the single‐guide RNA (sg RNA) scaffold of the vector. The sequences of oligos are listed in Table S3. The ligated plasmid was transformed into Stbl3 bacteria, plated with selection pressure, and the single colonies obtained were expanded prior to plasmid extraction using midi‐prep kit (Macherey‐Nagel). The correct insertion of the oligos was later confirmed by Sanger sequencing.

For the generation of virus, HEK293T cells were transfected with the constructs and lentiCRISPR v2 vector using the TransIT®‐293 transfection reagent (Mirus Bio, Madison, WI, USA), according to the manufacturer’s protocol. Viruses with negative control vector or constructed vector were harvested after 24–48 h. MDA‐MB‐231/A cells were infected with the viruses and selected using puromycin (Gibco). Produced MDA‐MB‐231/A stable cell line was cultured in DMEM (WelGENE Inc.) with 10% FBS (Gibco) and puromycin (1–10 μg·mL^−1^; Gibco).

### 2.19 Statistical analysis

One‐tailed *t*‐test was performed using graphpad Prism 5 software (GraphPad, La Jolla, CA, USA). Values have been expressed as the means ± SDs. *P* < 0.05 was considered statistically significant (*P < 0.05; **P < 0.01; ***P < 0.001). All experimental data are representative of at least three independent experiments.

## 3 Results

### 3.1 Different patterns of autophagy in breast CSCs

**Fig. 1:**
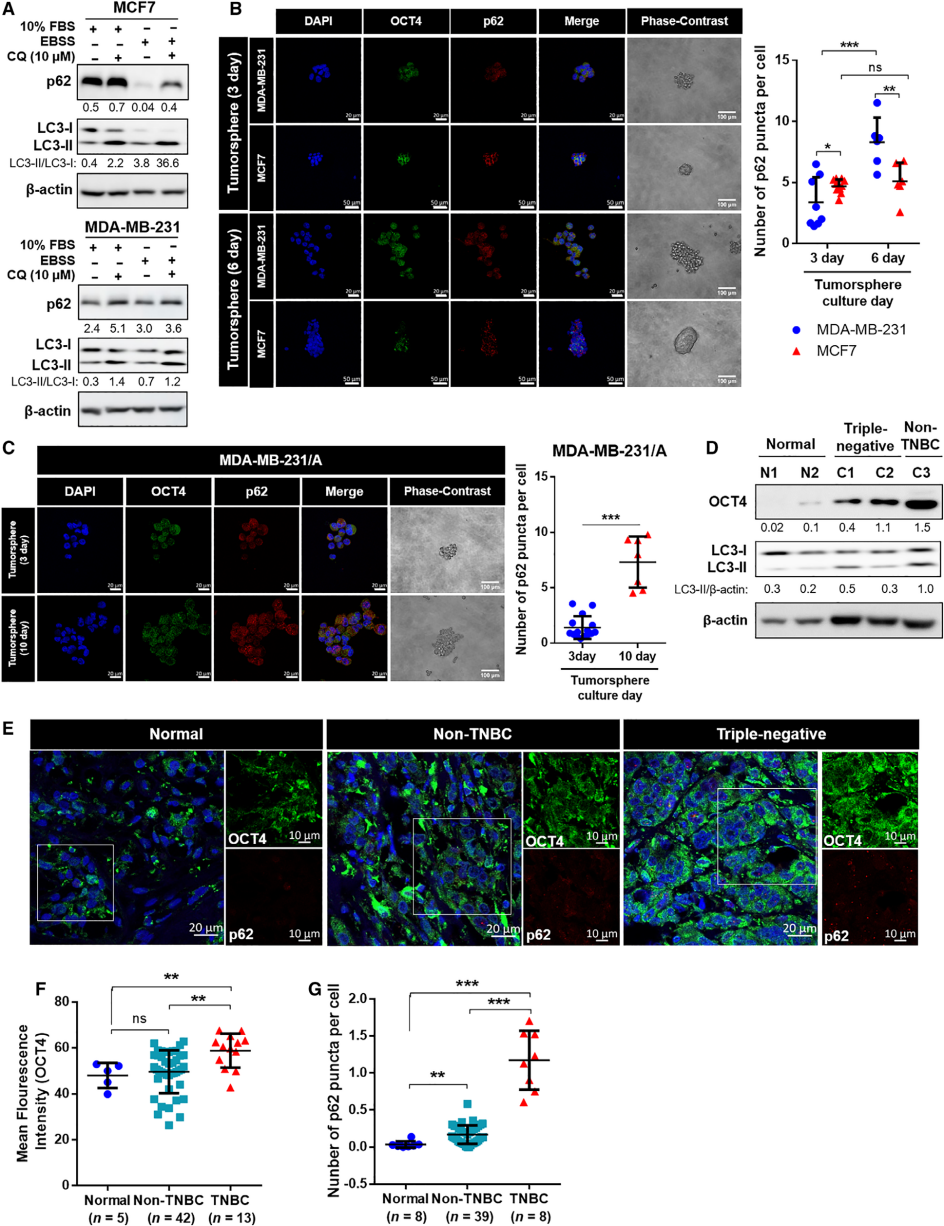
The expression of autophagy flux and cancer stemness in breast CSCs. (A) The expression level of LC3 and p62 in breast cancer cells. EBSS, Earle's balanced salt solution; CQ, chloroquine. (B,C) Immunocytochemistry staining of MDA‐MB‐231, MCF7, and MDA‐MB‐231/A tumorspheres for detection of OCT4 and p62 (magnification: ×40) and phase‐contrast images of tumorspheres (magnification: ×20). Nuclei were counterstained with DAPI. The number of p62 puncta was normalized with the number of DAPI‐stained nuclei. Scale bar, 20 μm (fluorescence images of MDA‐MB‐231 and MDA‐MB‐231/A), 50 μm (fluorescence images of MCF7), 100 μm (phase‐contrast images). Data were presented as mean ± SD of three independent experiments. Statistical analyses were performed with one‐tailed student’s *t*‐test (*P < 0.05, **P < 0.01, ***P < 0.001; ns, nonsignificant difference). (D) The expression levels of OCT4 and LC3 in normal breast and breast cancer tissues. (E) Representative images of immunofluorescence staining of breast cancer tissues (magnification: ×40, zoom: 2.0). The scale bar for magnified images equals 10 μm. Nuclei were counterstained with DAPI. Scale bar, 20 μm. (F) Mean fluorescence intensity of OCT4 in normal breast (*n* = 5), non‐TNBC (luminal and HER2; *n* = 42) and TNBC tissues (*n* = 13). (G) The expression level of p62 in normal breast (*n* = 8), non‐TNBC (*n* = 39), and TNBC tissues (*n* = 8). The numbers of p62 puncta were normalized with the number of DAPI‐stained nuclei. (F,G) Each dot represents one patient and intensity value of about 150 cells was measured on average per patient. The normal tissues used in the experiment are not paired with cancer tissues. Data were presented as mean ± SD. Statistical analyses were performed with one‐tailed Student’s *t*‐test (**P < 0.01, ***P < 0.001; ns, nonsignificant difference).

First, we aimed to investigate the difference of autophagy flux in two subtypes of breast CSCs. MDA‐MB‐231 (TNBC) and MCF7 (luminal), two of the most well‐known and widely studied breast cancer cell lines, were selected for comparison. Alterations of autophagy genes have been observed in them, suggesting the significant effects of autophagy on characteristics of cancer cells [13, 14]. To establish stem‐like groups of breast cancer cells, MDA‐MB‐231 and MCF7 cells were grown in tumorspheres [27] and compared to cells grown in monolayer. When subjected to a starved conditions, induced by treatment with EBSS, MDA‐MB‐231 cells showed less progression of autophagy flux, as there was less conversion of LC3‐I to LC3‐II. The expression of p62 also showed no significant difference between basal and starved conditions in MDA‐MB‐231. The accumulation of p62 in MCF7 was significantly higher when treated with chloroquine (CQ), which suggests that the amount of basal autophagosome in MCF7 is greater than that of MDA‐MB‐231 (Fig. 1A). These results indicate that autophagy does indeed show differences, depending on the subtypes of breast CSCs.

To further observe autophagy in breast CSCs, the expression level of another widely used autophagy marker, p62, was measured by ICC, in addition to the expression level of cancer stemness marker, OCT4 [28, 29]. An increase in the expression levels of p62 was also observed in the tumorsphere culture of MDA‐MB‐231 cells, which can be interpreted as an attenuation of the overall autophagy flux [30, 31], suggesting that autophagy was attenuated only in TNBC CSCs (Fig. 1B).

To investigate the precise relationship of cancer stemness and autophagy in TNBC CSCs, the MDA‐MB‐231/A cell line was included in the ICC analysis of tumorspheres. MDA‐MB‐231/A is the immortalized MDA‐MB‐231 cell line, obtained from mice tumors generated by the injection of MDA‐MB‐231 cells. Cancer cells retrieved from primary tumors are known to contain a higher population of CSCs [32]. Thus, MDA‐MB‐231/A is closer to the state of CSCs than MDA‐MB‐231. The expression of p62 showed increase in MDA‐MB‐231/A tumorspheres, as in MDA‐MB‐231, which also represents inhibition of autophagy (Fig. 1C). OCT4 expression was found to increase as the tumorspheres formed and grew, suggesting that the tumorspheres reliably acquired cancer stemness during their formation. (Fig. S1). Autophagy did not show attenuation in HER2 breast cancer tissue with high OCT4 expression (Fig. 1D). The immunofluorescence assay revealed that TNBC tissues showed high expression of p62 and OCT4, compared to normal and non‐TNBC tissues, suggesting that the attenuation of autophagy is related to cancer stemness of TNBC (Fig. 1E–G). These results suggest that autophagy is potentially negatively correlated with cancer stemness in TNBC. If autophagy acts as a tumor suppressor only in TNBC CSCs and not in other subtypes, TNBC will require a therapeutic approach that is distinct from other subtypes.

### 3.2 miR‐181a is upregulated in TNBC CSCs and directly regulates ATG5 and ATG2B

We attempted to investigate the mechanism by which autophagy affects cancer stemness in TNBC. As miRNA affects autophagy through the suppression of protein expression [33, 34, 35], we suggested the possibility that a specific miRNA attributes the cancer stemness and aggressiveness of TNBC by targeting autophagy molecules. miRNAs that are differentially expressed between MDA‐MB‐231 and MCF7 cells have been identified to delineate the differences between common breast cancer cells and CSCs [36]. The miRNA expression profile in tumorspheres was compared to that of parental cells grown in monolayer culture. This led to the identification of miRNAs that are upregulated in the MDA‐MB‐231 and MCF7 tumorspheres, compared to their parent cells [36]. Based on the above findings and in search for deregulated miRNAs in TNBC CSCs, we analyzed the list of miRNA identified at the dataset described above (GSE75396) [36] to find miRNAs upregulated only in MDA‐MB‐231 tumorspheres.

**Fig. 2:**
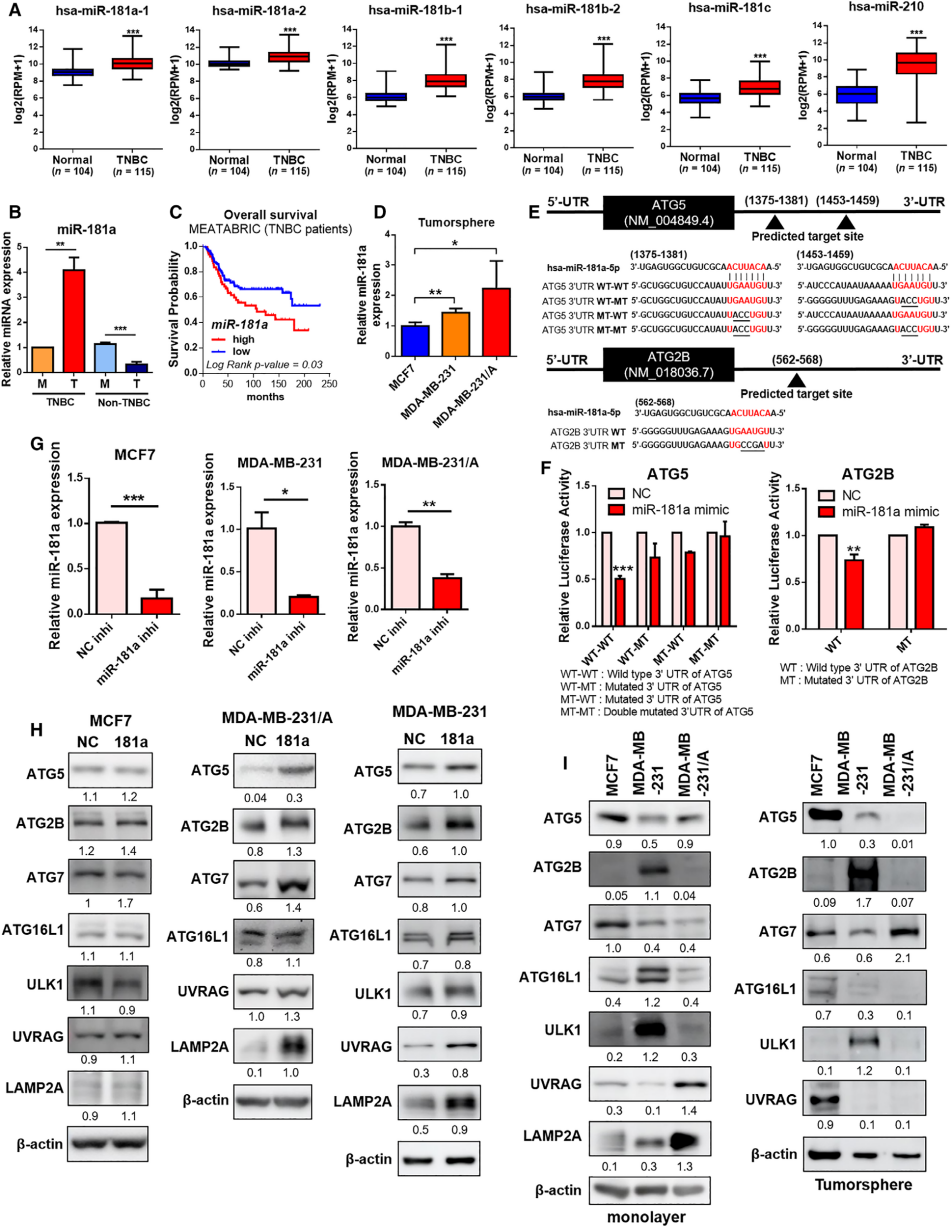
miR‐181a is upregulated in TNBC CSCs and directly targets autophagy genes. (A) The expression levels of six miRNAs between TNBC (*n* = 115) and normal tissues (*n* = 104). MOBCdb was used to confirm expression levels. Statistical analyses were performed with one‐tailed Student’s *t*‐test (***P < 0.001). (B) The expression levels of miR‐181a in TNBC (MDA‐MB‐231) and non‐TNBC (MCF7) cells. M, monolayer; T, tumorsphere. Data were presented as mean ± SD of three independent experiments. Statistical analyses were performed with one‐tailed Student’s *t*‐test (**P < 0.01, ***P < 0.001). (C) Kaplan–Meier analysis of METABRIC datasets showing overall survival of TNBC patients. *P*‐value was calculated using the log‐rank test. Patients were stratified into ‘low (blue)’ and ‘high (red)’ miR‐181a expression groups based on the auto‐selected best cut‐off. (D) miR‐181a expression level in breast cancer tumorspheres. Data were presented as mean ± SD of three independent experiments. Statistical analyses were performed with one‐tailed Student’s *t*‐test (*P < 0.05, **P < 0.01). (E) The sequence alignment of miR‐181a and the 3′ UTRs of ATG5 and ATG2B containing miRNA‐binding sites. (F) Luciferase activities of the wild‐type or mutant 3′ UTR constructs of ATG5 and ATG2B in HEK293T cells 24–48 h after transfection with miR‐181a mimic or negative control. NC, negative control mimic. Data were presented as mean ± SD of three independent experiments. Statistical analyses were performed with one‐tailed Student’s *t*‐test (**P < 0.01, ***P < 0.001). (G) qRT‐PCR analysis showing the miR‐181a expression level in breast cancer cells after transfection with miR‐181a inhibitor or negative control. NC inhi, negative control inhibitor; miR‐181a inhi, miR‐181a inhibitor. Data of MCF7 were presented as mean ± SD of five independent experiments. Data of MDA‐MB‐231 and MDA‐MB‐231/A were presented as mean ± SD of three independent experiments. Statistical analyses were performed with one‐tailed Student’s *t*‐test (*P < 0.05, **P < 0.01, ***P < 0.001). (H) Protein expression levels of autophagy genes in breast cancer cells transfected with negative control (NC) or miR‐181a inhibitor (181a). (I) Protein expression levels of autophagy genes in breast cancer cells grown in monolayer or tumorspheres.

Based on analysis of GSE75396 [36], 33 miRNAs showed increased expression in tumorspheres relative to the parental cells. Out of these 33 miRNAs, 23 were upregulated only in MDA‐MB‐231 tumorspheres, not in MCF7 tumorspheres. To identify how the expression of these miRNAs differ in normal and breast cancer patients, the list of 23 miRNAs went through TCGA miRNA analysis provided by UALCAN [37] and MOBCdb [38] (Fig. S2). Eight miRNAs were found to be upregulated in tissues from TNBC patients compared to adjacent normal patients, and six of the eight miRNAs were upregulated in TNBC compared with luminal breast cancer. Through the analysis of TCGA data using MOBCdb [38], the expression levels of these six miRNAs between normal and TNBC tissues were confirmed; of the six miRNAs, miR‐181a‐1, miR‐181a‐2, miR‐181b‐1, miR‐181b‐2, miR‐181c, and miR‐210 had higher expression levels in TNBC tissues than in normal tissues (Fig. 2A). miR‐181a was actually found to be upregulated in the TNBC tumorspheres, compared to that in the cells cultured in monolayer, whereas upregulation of miR‐181a was not observed in non‐TNBC tumorspheres (Fig. 2B). Three‐dimensional spheroid formation of breast cancer cells was confirmed in the anchorage‐independent environment of tumorsphere culture (Fig. S3A). High expression levels of OCT4 and SOX2 confirmed the enrichment of CSCs in the tumorspheres (Fig. S3B) [28, 29, 39]. Kaplan–Meier analysis of METABRIC datasets also revealed that a low level of overall survival was observed in the TNBC patient group with high miR‐181a expression (Fig. 2C). For these reasons, the present study was carried forward with a focus on the upregulation of only miR‐181a in the CSC population.

TNBC tumorspheres were found to be more enriched with CSCs, this was confirmed by determining the expression of OCT4 and SOX2 (Fig. S3C). MDA‐MB‐231/A cells also displayed increased expression of miR‐181a in tumorspheres, compared to parental cells (Fig. S4A). The basal expression level of miR‐181a was higher in MDA‐MB‐231/A than in MDA‐MB‐231 (Fig. S4B), and in the CSC population, miR‐181a expression level was found to be higher in TNBC cells than in MCF7 cells (Fig. 2D). These results so far suggest that higher expression of miR‐181a in TNBC may be correlated with cancer stemness.

To find whether autophagy is involved in the relationship between miR‐181a and cancer stemness, we attempted to investigate whether miR‐181a affects autophagy. Since miRNAs regulate the expression of target genes by binding to their 3′ UTRs [40], TargetScan was used to look for autophagy genes containing potential miRNA regulatory elements (MREs) for miR‐181a in their 3′ UTRs. Additionally, HADb, a database containing known autophagy genes in humans was used for comparative analysis with TargetScan. The analysis showed that 66 genes from HADb were found to have miR‐181 MREs in their 3′ UTRs (Table S4).

miR‐181a binds directly to ATG5 in luminal breast cancer, and the regulation of ATG5 by miR‐181a affects autophagy flux in MCF7 [41]. ATG2B is also regulated by miRNA and thus affects autophagy in NSCLC and chronic lymphocytic leukemia cells [42, 43]. Therefore, we selected ATG5 and ATG2B out of the 66 genes as candidate targets of miR‐181a. To confirm whether miR‐181a actually binds to the 3′ UTRs of ATG5 and ATG2B, a dual‐luciferase assay was performed using ATG5 and ATG2B 3′ UTR constructs, which contain the binding seed sequence for miR‐181a (Fig. 2E). The luciferase activities of wild‐type ATG5 and ATG2B 3′ UTRs were found to be highly attenuated upon miR‐181a overexpression, suggesting direct binding between miR‐181a and the two genes (Fig. 2F). In order to observe the effect of the inhibition of miR‐181a, miR‐181a inhibitor was ectopically transfected to breast cancer cells, and it was confirmed that the expression of miR‐181a was significantly inhibited (Fig. 2G). Among the miRNAs increased in TNBC tissue, the inhibitor specifically reduced the expression of miR‐181a, with the exception of miR‐181c which showed exceptionally low expression in the breast cancer (Fig. S5). When miR‐181a was inhibited, the expression of ATG5 and ATG2B was increased in TNBC cells, while the expression of these genes did not show significant changes in MCF7 cells (Fig. 2G,H). Additionally, it was confirmed that the expression levels of ATG5 and ATG2B were higher in MDA‐MB‐231 cells than in MDA‐MB‐231/A cells grown in tumorspheres compared to that in monolayer (Fig. 2I). Based on these results, it can be concluded that miR‐181a regulates the expression of ATG5 and ATG2B by directly binding to the 3′ UTRs of ATG5 and ATG2B in TNBC.

### 3.3 Inhibition of miR‐181a promotes autophagy flux and attenuates cancer stemness in TNBC

**Fig. 3:**
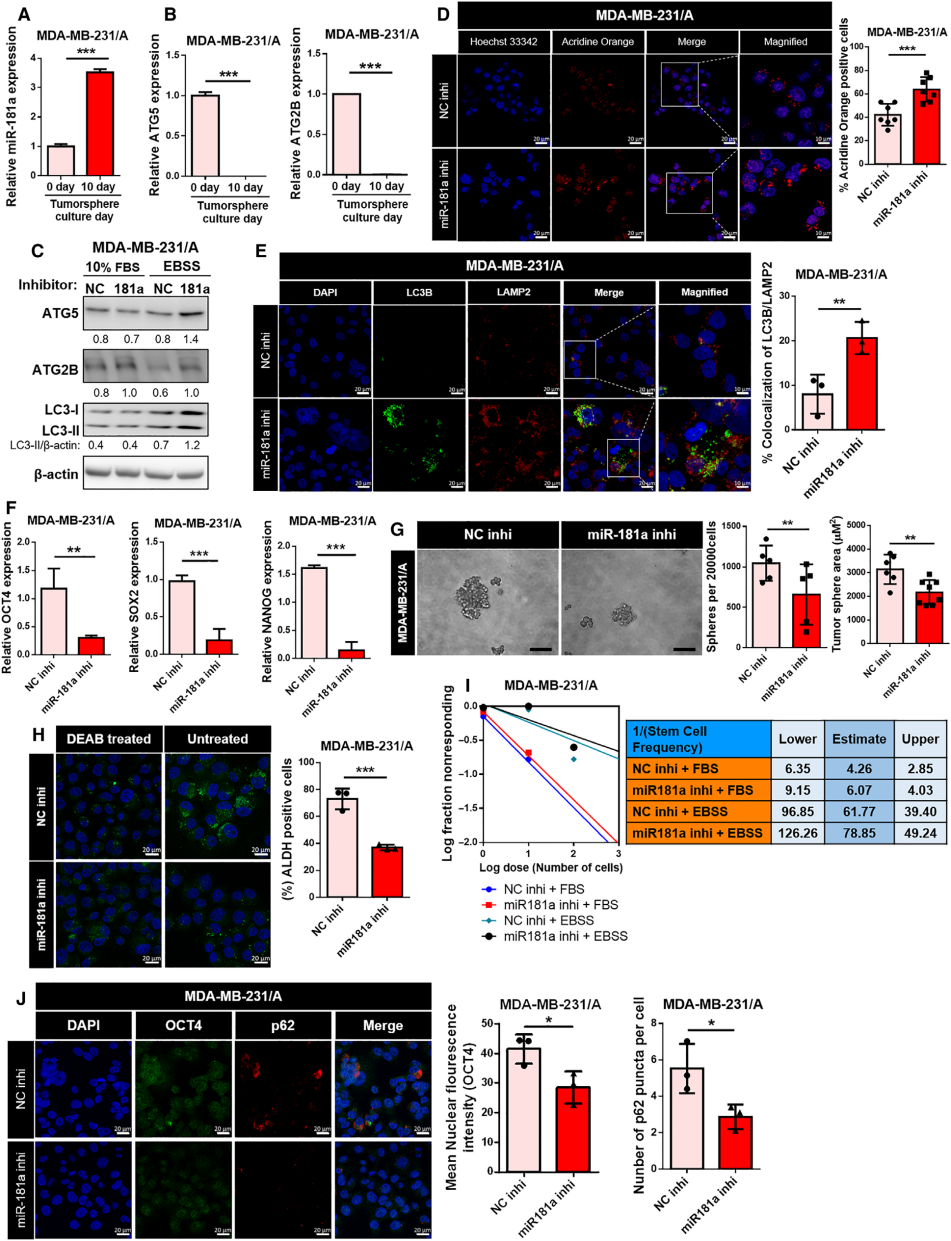
Inhibition of miR‐181a led to changes in autophagy and cancer stemness. (A) Expression levels of miR‐181a in MDA‐MB‐231/A tumorspheres were assessed using Taqman qRT‐PCR on days 0–10 of tumorsphere culture. Data were presented as mean ± SD of three independent experiments. Statistical analyses were performed with one‐tailed Student’s *t*‐test (***P < 0.001). (B) ATG5 and ATG2B mRNA levels in MDA‐MB‐231/A tumorspheres. Data were presented as mean ± SD of three independent experiments. Statistical analyses were performed with one‐tailed Student’s *t*‐test (***P < 0.001). (C) Expression levels of ATG5, ATG2B, and LC3 proteins in cells transfected with miR‐181a inhibitor. NC, negative control. (D) Confocal microscopy of acridine orange staining in MDA‐MB‐231/A tumorspheres (left, magnification: ×40, zoom: 2.0). The scale bar for magnified images equals 10 μm. Nuclei were counterstained with Hoechst 33342. Scale bar, 20 μm. The percentage of MDA‐MB‐231/A cells containing acridine orange puncta is shown (right). The number of nuclei was used to normalize the values. Data were presented as mean ± SD. Statistical analyses were performed with one‐tailed Student’s *t*‐test (***P < 0.001). (E) Confocal microscopy of LC3B and LAMP2 staining in tumorspheres (left, magnification: ×40, zoom: 2.0). The scale bar for magnified images equals 10 μm. Nuclei were counterstained with DAPI. Scale bar, 20 μm. Colocalization of LC3B and LAMP2 puncta was quantified, and normalized with the number of all measured puncta (right). Data were presented as mean ± SD. Statistical analyses were performed with one‐tailed Student’s *t*‐test (**P < 0.01). (F) The mRNA levels of OCT4, SOX2, and NANOG in MDA‐MB‐231/A cells after transfection with miR‐181a inhibitors were measured using qRT‐PCR. Data were presented as mean ± SD of four independent experiments. Statistical analyses were performed with one‐tailed Student’s *t*‐test (**P < 0.01, ***P < 0.001). (G) Representative images of MDA‐MB‐231/A tumorspheres transfected with miR‐181a inhibitors (left, magnification: ×20). Scale bar, 100 μm. The number and sizes of tumorspheres were quantified (right). Data were presented as mean ± SD. Statistical analyses were performed with one‐tailed Student’s *t*‐test (**P < 0.01). (H) MDA‐MB‐231/A cells transfected with miR‐181a inhibitor were stained with Aldefluor reagent (left). Cells treated additionally with DEAB (Aldefluor inhibitor) were used as a negative control. Nuclei were counterstained with Hoechst 33342 (Hoechst 33342; blue). Scale bar, 20 μm. The percentage of DEAB‐untreated MDA‐MB‐231/A cells containing ALDH (Aldefluor; green) puncta is shown (right). The number of nuclei was used to normalize the values. Data were presented as mean ± SD. Statistical analyses were performed with one‐tailed Student’s *t*‐test (***P < 0.001). (I) Limiting dilution assay performed on MDA‐MB‐231/A cells transfected with miR‐181a inhibitors. Stem cell frequency was calculated using extreme limiting dilution assay analysis. (J) Immunocytochemistry staining for detection of p62 and OCT4 in MDA‐MB‐231/A cells transfected with miR‐181a inhibitor (left, magnification: ×40). Nuclei were counterstained with DAPI. Scale bar, 20 μm. Mean nuclear intensity of OCT4 fluorescence is shown (right). The number of p62 puncta was normalized with the number of DAPI‐stained nuclei (right). Data were presented as mean ± SD. Statistical analyses were performed with one‐tailed Student’s *t*‐test (*P < 0.05).

Based on the previous results, miR‐181a may have an effect on autophagy flux by regulating two of the early autophagy genes. miR‐181a expression was found to increase from the early to late days of tumorsphere culture of TNBC cells (Fig. 3A and S6A). The expression of miR‐181a did not change significantly in MCF7 tumorspheres (Fig. S6B). The increase in the levels of miR‐181a brought about corresponding changes in the expression levels of ATG5 and ATG2B (Fig. 3B and S6C). To identify the precise effect of miR‐181a on autophagy flux of TNBC CSCs, MDA‐MB‐231/A cells were used, as it possess more stem‐like properties compared to MDA‐MB‐231. The extent of increase in ATG5 and ATG2B levels upon inhibition of miR‐181a became more explicit in the starved condition (Fig. 3C). Also, when miR‐181a was inhibited, there was an increase observed in the autolysosome formation which was detected by acridine orange staining (Fig. 3D) [44]. The colocalized puncta of LC3B‐LAMP2 also increased when miR‐181a was inhibited, suggesting that inhibition of miR‐181a promoted fusion of autophagosome (LC3B as the marker) with lysosome (LAMP2 as the marker) and the progress of autophagy to its late stages (Fig. 3E) [45, 46].

Cancer stemness markers, OCT4, SOX2, and NANOG [47], were found to be significantly decreased upon miR‐181a inhibition (Fig. 3F). In addition, tumorsphere formation ability of TNBC cells transfected with the miR‐181a inhibitor was found to be impaired (Fig. 3G). In various types of tumors, aldehyde dehydrogenase (ALDH) is associated with cancer stem cell phenotype including tumor‐initiating ability [48, 49, 50]. We found that miR181a inhibition decreased ALDH‐positive cells, suggesting the attenuation of cancer stemness (Fig. 3H). A decrease in the stem cell frequency in cells transfected with the miR‐181a inhibitor was also observed in limiting dilution assay (Fig. 3I). A reduction in the number of p62 puncta after the inhibition of miR‐181a indicated degradation of autophagosomal cargo proteins, suggesting the promotion of autophagy, while a decrease in nuclear OCT4 expression suggested the attenuation of cancer stemness (Fig. 3J). So far, changes in both autophagy flux and cancer stemness were observed when the expression of miR‐181a changed. CCK‐8 assay revealed that inhibition of miR‐181a itself had no significant effect on MDA‐MB‐231/A cell proliferation (Fig. S7), indicating that miR‐181a inhibition decreases cancer stemness without affecting cell viability. These results suggest the possibility that miR‐181a plays a role in the relationship between tumor‐suppressive autophagy and cancer stemness in TNBC CSCs.

### 3.4 The promotion of autophagy by curcumin negatively affects cancer stemness in TNBC

If autophagy affects the cancer stemness of TNBC through the action of miR‐181a, it is essential to investigate whether the regulation of autophagy affects cancer stemness by controlling the target genes of miR‐181a. Previously, autophagy flux was found to be inversely proportional to the expression of cancer stemness markers (Fig. 3). Judging from the results of the present study and findings from previous reports, autophagy seems to participate in the suppression of cancer stemness. To precisely verify the relation between upregulation of miR‐181a and attenuation of autophagy in TNBC CSCs, the effects of ATG5 and ATG2B were replicated in MDA‐MB‐231/A by restoring autophagy at a molecular level. Thus, in the present study, curcumin, a natural compound known for its autophagy‐inducing and anti‐tumor effects [51], was selected to function as an autophagy inducer to recover the downregulated autophagy flux in TNBC CSCs.

**Fig. 4:**
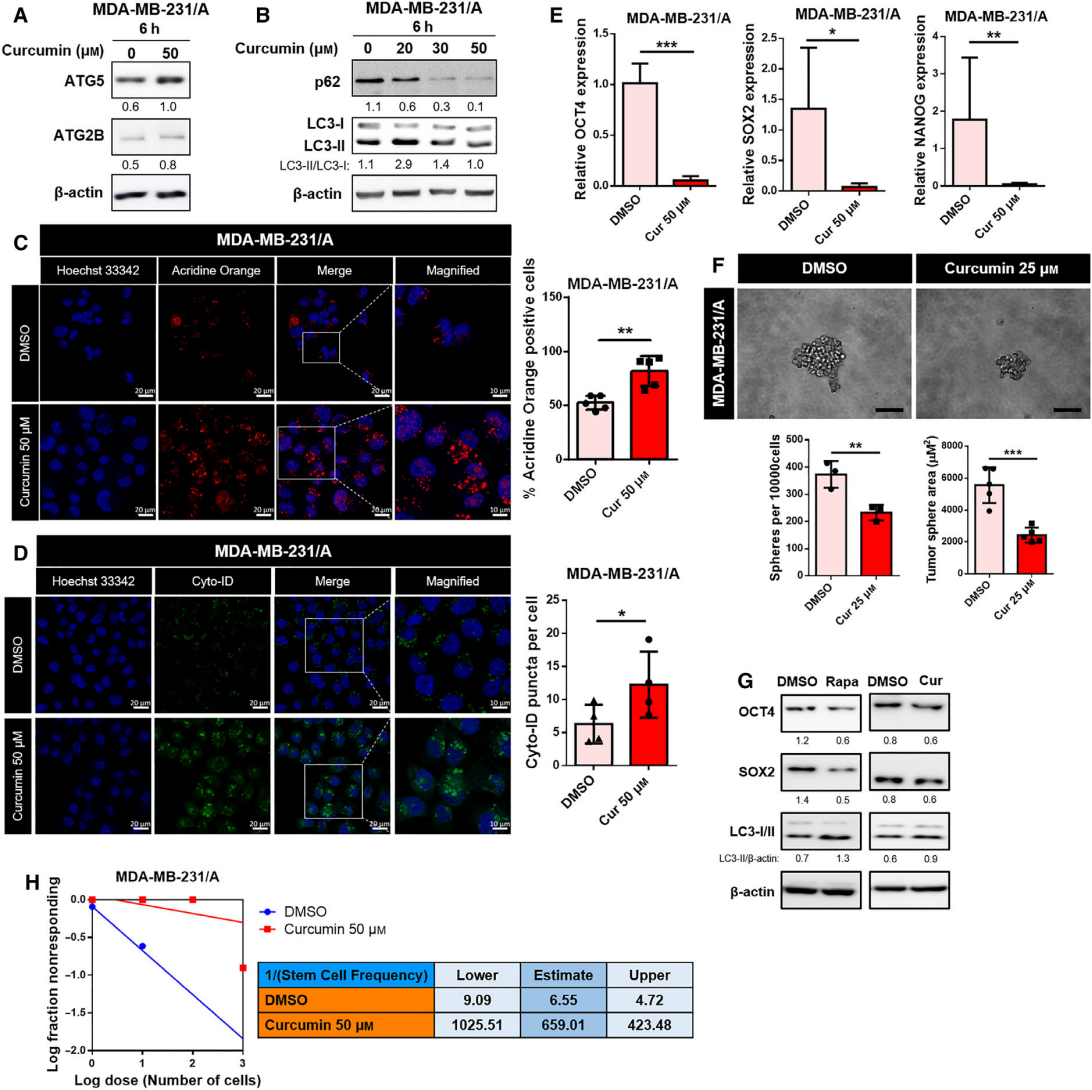
Recovery of autophagy by curcumin inhibits cancer stemness in TNBC. (A) Protein levels of ATG5 and ATG2B in curcumin‐treated MDA‐MB‐231/A cells. (B) Treatment of MDA‐MB‐231/A cells with different concentrations of curcumin. (C) Confocal microscopy of acridine orange staining in curcumin‐treated MDA‐MB‐231/A cells (left, magnification: ×40, zoom: 2.0). The scale bar for magnified images equals 10 μm. Nuclei were counterstained with Hoechst 33342. Scale bar, 20 μm. The percentage of MDA‐MB‐231/A cells containing acridine orange puncta is shown (right). The number of nuclei was used to normalize the values. Data were presented as mean ± SD. Statistical analyses were performed with one‐tailed Student’s *t*‐test (**P < 0.01). (D) Confocal microscopy of curcumin‐treated MDA‐MB‐231/A cells stained with Cyto‐ID dye (left, magnification: ×40, zoom: 2.0). The scale bar for magnified images equals 10 μm. Nuclei were counterstained with Hoechst 33342. Scale bar, 20 μm. The number of Cyto‐ID puncta was normalized with the number of nuclei (right). Data were presented as mean ± SD. Statistical analyses were performed with one‐tailed Student’s *t*‐test (*P < 0.05). (E) mRNA expression of cancer stemness markers in curcumin‐treated MDA‐MB‐231/A cells. Data were presented as mean ± SD of three independent experiments. Statistical analyses were performed with one‐tailed Student’s *t*‐test (*P < 0.05, **P < 0.01, ***P < 0.001). (F) Representative images of MDA‐MB‐231/A tumorspheres treated with curcumin (left, magnification: ×20). Scale bar, 100 μm. The number and sizes of tumorspheres were quantified (below). Data were presented as mean ± SD. Statistical analyses were performed with one‐tailed Student’s *t*‐test (**P < 0.01, ***P < 0.001). (G) Protein levels of OCT4, SOX2, and LC3 in rapamycin or curcumin‐treated MDA‐MB‐231/A cells. (H) Limiting dilution assay performed on MDA‐MB‐231/A cells treated with curcumin. Stem cell frequency was calculated using extreme limiting dilution assay analysis.

Curcumin was found to stimulate the expression of ATG5 and ATG2B in MDA‐MB‐231/A (Fig. 4A), and treatment with different concentrations of curcumin was found to induce autophagy flux in TNBC cells (Fig. 4B and S8A). The concentration of curcumin that induced autophagy flux in MDA‐MB‐231/A was also found to stimulate the formation of autolysosomes (Fig. 4C). Cyto‐ID® staining further verified that treatment of curcumin induced the formation of late autophagic vacuoles (Fig. 4D). The treatment of curcumin caused a decrease in the gene expression of cancer stemness markers and the formation of tumorspheres (Fig. 4E,F and Fig. S8B). Treatment of curcumin did not change the expression of miR‐181a, thus indicating that curcumin does not affect autophagy flux of MDA‐MB‐231/A by regulating the expression of miR‐181a (Fig. S8C). Consistent with curcumin effect on tumorsphere formation, rapamycin treatment or ATG5 overexpression to induce autophagy flux also decreased tumorsphere formation of MDA‐MB‐231/A (Fig. S9A,B). Additionally, we show that protein expression of stemness markers, OCT4 and SOX2, were decreased under treatment of rapamycin or curcumin in MDA‐MB‐231/A (Fig. 4G), indicating that stemness markers are considered as autophagy target. These results suggest that autophagy inhibited cancer stemness via regulating stemness marker in TNBC cells. In limiting dilution assay, stem cell frequency in cells treated with curcumin decreased (Fig. 4H), thus suggesting that the recovery of autophagy by curcumin negatively affects the cancer stemness.

### 3.5 Tumor‐suppressive effect of curcumin is enhanced in miR‐181a‐deficient TNBC cells

**Fig. 5:**
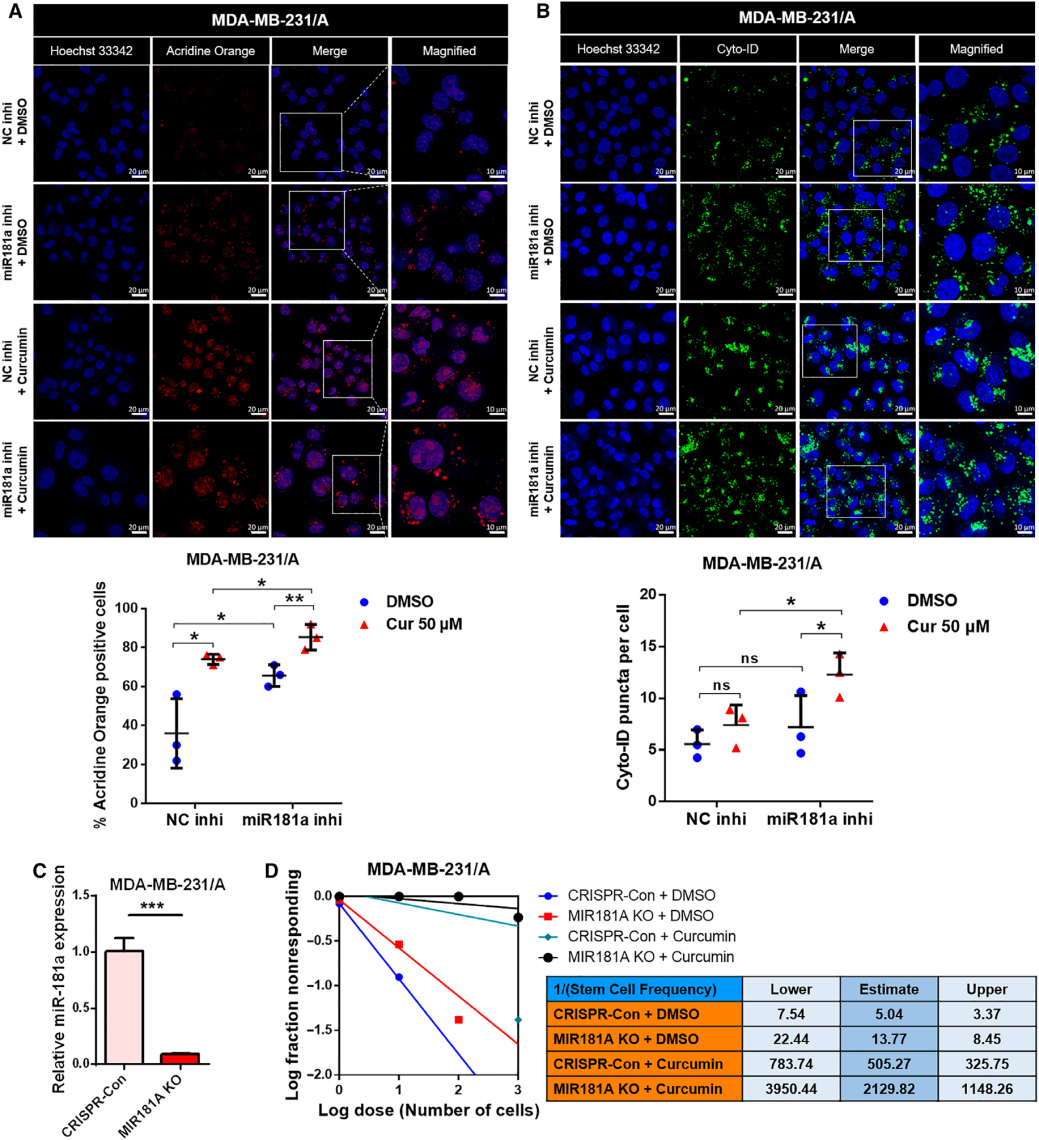
Treatment with curcumin enhances the suppression of cancer stemness by miR‐181a inhibitor. (A) Confocal microscopy of acridine orange staining in MDA‐MB‐231/A cells treated with curcumin following miR‐181a transfection. (Magnification: ×40, zoom: 2.0). The scale bar for magnified images equals 10 μm. Nuclei were counterstained with Hoechst 33342. Scale bar, 20 μm. The percentage of MDA‐MB‐231/A cells containing acridine orange puncta is shown. The number of nuclei was used to normalize the values. Data were presented as mean ± SD. Statistical analyses were performed with one‐tailed Student’s *t*‐test (*P < 0.05, **P < 0.01). (B) Confocal microscopy of autophagic MDA‐MB‐231/A cells stained with Cyto‐ID dye following miR‐181a transfection and curcumin treatment (magnification: ×40, zoom: 2.0). The scale bar for magnified images equals 10 μm. Nuclei were counterstained with Hoechst 33342. Scale bar, 20 μm. The number of Cyto‐ID puncta was normalized with the number of nuclei. Data were presented as mean ± SD. Statistical analyses were performed with one‐tailed Student’s *t*‐test (*P < 0.05; ns, nonsignificant difference). (C) qRT‐PCR analysis showing the miR‐181a expression levels in MDA‐MB‐231/A stable KO cells. Data were presented as mean ± SD of three independent experiments. Statistical analyses were performed with one‐tailed Student’s *t*‐test (***P < 0.001). (D) Limiting dilution assays performed on curcumin‐treated MDA‐MB‐231/A stable miR‐181a‐KO cells. Stem cell frequency was calculated using extreme limiting dilution assay analysis.

To confirm whether miR181a inhibition induces tumor‐suppressive effect of curcumin, we examined the effect of curcumin treatment on autophagy and cancer stemness in miR‐181a‐deficient TNBC cells. We found that curcumin treatment further promoted the formation of autolysosomes and late autophagic vacuoles, in addition to the effects of the inhibition of miR‐181a (Fig. 5A,B). In order to observe the long‐term changes in cancer stemness using the limiting dilution assay, an MDA‐MB‐231/A stable cell line with continuous low expression of miR‐181a was produced (MIR181A‐KO) (Fig. 5C and Fig. S10). As shown in Fig. S10, ATG2B, ATG5, and autophagy flux were increased, while OCT4 was decreased in miR‐181a KO cells compared to that in controls. A decrease in the CSC‐initiating frequency of MDA‐MB‐231/A cells was observed when the expression of miR‐181a was stably inhibited (Fig. 5D). Curcumin lowered CSC‐initiating frequency in TNBC, as well as the inhibition of miR‐181a (Fig. 5D). When the effect of miR‐181a inhibition and curcumin treatment overlapped, cancer stemness was further reduced in MDA‐MB‐231/A stable KO cells (Fig. 5D). These results indicated that the suppression of miR‐181a further enhanced the tumor‐suppressive effects of curcumin. Thus, it can be concluded that the regulation of autophagy through miR‐181a has clinically meaningful suppressive effects on cancer stem cells.

## 4 Discussion

Since TNBC is enriched with the CSC population, which serve as influencing factors in its resistance to chemotherapy, CSC‐targeted therapy is an important process in treating cancer [52]. Recently, many studies have applied autophagy for CSC‐targeted therapy; autophagy itself has been shown to play a dual role in cancer, as a tumor suppressor and a tumor promoter [15]. Therefore, autophagy acts as a double‐edged sword for tumor cells. Autophagy genes have frequently been found to be mono‐allelically deleted, silenced, or mutated in human tumors, resulting in an environment of increased oxidative stress that is conducive to DNA damage, genomic instability, and tumor progression [53]. Cancer tissues have been shown to display reduced autophagy as compared to their normal counterparts, suggesting that autophagy serves as an *in vivo* tumor suppressor [54].

Dysregulation of miRNA has been observed in various types of human cancer [55]. Numerous studies revealed that miRNAs play important roles in autophagy, as several target genes of miRNAs have been found to be actively involved in the autophagy pathway [33, 34, 35, 56]. In a mammary epithelial cell line, autophagy promotes the survival of cells detached from surfaces, and miR‐181a promotes anoikis by suppressing autophagy [57], but the role of autophagy in breast cancer varies by subtype [13]. In cancer stem cells, autophagy is known to display a more complex status [58]. It has been found that miR‐181a regulates autophagy and sustains cellular survival in various cancers [59, 60]. Moreover, the inhibition of miR‐181a was observed to have therapeutic effects on gastric cancer [61].

Curcumin is a natural compound known for its anticancer effects in various cancers and antiproliferative effects on human tumor cell lines [62, 63]. Curcumin has been shown to upregulate the expression of autophagy‐related proteins, including ATG5 and ATG2B and induce overall autophagy flux in human cancers [64, 65]. Autophagy modulation in CSCs is an emerging strategy for cancer therapy, involving the use of drugs or natural compounds that regulate autophagy flux. Many of the clinical drugs and natural compounds induce autophagy to accelerate cancer cell death, as a means of suppressing cancer growth [53].

By demonstrating that downregulation of autophagy occurs specifically in TNBC with high expression of cancer stemness marker, the present study intended to suggest the role of autophagy as a potential tumor suppressor in TNBC CSCs. Also, our data collectively led us to the conclusion that the upregulation of miR‐181a results in the downregulation of its target genes ATG5 and ATG2B in TNBC CSCs. This leads to the attenuation of the tumor‐suppressive autophagy, resulting in the stable maintenance of cancer stemness. The promotion of autophagy by curcumin, which increases the expression of the target gene of miR‐181a, could be used as a means of tumor suppression and a treatment for TNBC. Inhibition of miR‐181a and treatment of curcumin compensate for the downregulation of autophagy flux and restore it; the tumor‐suppressive autophagy then results in the inhibition of cancer stemness. Therefore, based on the results in this study, we present miR‐181a as a potential target of therapy and curcumin as a potential treatment for TNBC.

## 5 Conclusions

Taken together, this study identified the tumor‐suppressive effect of autophagy regulated by miR‐181a on TNBC stemness, which could provide a new evidence of miRNA‐regulated autophagy and potential therapeutic approach for TNBC.

## Conflict of interest

The authors declare no conflict of interest.

### Peer review

The peer review history for this article is available at https://publons.com/publon/10.1002/1878‐0261.13180.

## Author contributions

JWP and YK performed the experiments, analyzed the data, and prepared the manuscript. SBL, CWO, and EJL contributed to data and methods. JYK performed the experiments, analyzed the data, and prepared the revised manuscript. JHP contributed to conceptualization and funding acquisition and discussed the manuscript.

## Consent for publication

Not applicable.

## Supporting information


**Fig. S1**. The expression of OCT4 in breast cancer tumorspheres.
**Fig. S2**. The expression levels of miRNAs upregulated in MDA‐MB‐231 tumorspheres between normal and TNBC tissues.
**Fig. S3**. The expression of cancer stem cell properties of breast cancer tumorspheres.
**Fig. S4**. miR‐181a expression in MDA‐MB‐231 and MDA‐MB‐231/A cells.
**Fig. S5**. The specificity of miR‐181a inhibitor on breast cancer cells.
**Fig. S6**. The expression of miR‐181a and autophagy target genes in breast cancer tumorspheres.
**Fig. S7**. The effect of miR‐181a inhibition on the viability of MDA‐MB‐231/A cells.
**Fig. S8**. The effects of curcumin on autophagy flux and cancer stemness of TNBC cells.
**Fig. S9**. The effects of ATG5 overexpression or rapamycin treatment on autophagy flux and tumorsphere of MDA‐MB‐231/A cells.
**Fig. S10**. Protein expression of autophagy and stemness marker in miR181a KO MDA‐MB‐231/A cells.

**Table S1**. qRT‐PCR primer sequences for each gene.
**Table S2**. The sequences of primers to amplify the 3’UTRs of the genes.
**Table S3**. The sequences of oligos for CRISPR‐knockout of miR‐181a.
**Table S4**. Target prediction of miR‐181a using TargetScan and HADb.

## Data Availability

The data that support the findings of this study are available on request from the corresponding author.
