# Supplementary material for: Autophagy inhibits cancer stemness in triple‐negative breast cancer via miR‐181a‐mediated regulation of ATG5 and/or ATG2B
Source: Mol Oncol. 2022 Jan 26;16(9):1857–75. doi: 10.1002/1878-0261.13180 (PMC9067148; doi:10.1002/1878-0261.13180)
Supplement: Supplementary file 1 — Fig. S1. The expression of OCT4 in breast cancer tumorspheres. Fig. S2. The expression levels of miRNAs upregulated in MDA‐MB‐231 tumorspheres between normal and TNBC tissues. Fig. S3. The expression of cancer stem cell properties of breast cancer tumorspheres. Fig. S4. miR‐181a expression in MDA‐MB‐231 and MDA‐MB‐231/A cells. Fig. S5. The specificity of miR‐181a inhibitor on breast cancer cells. Fig. S6. The expression of miR‐181a and autophagy target genes in breast cancer tumorspheres. Fig. S7. The effect of miR‐181a inhibition on the viability of MDA‐MB‐231/A cells. Fig. S8. The effects of curcumin on autophagy flux and cancer stemness of TNBC cells. Fig. S9. The effects of ATG5 overexpression or rapamycin treatment on autophagy flux and tumorsphere of MDA‐MB‐231/A cells. Fig. S10. Protein expression of autophagy and stemness marker in miR181a KO MDA‐MB‐231/A cells. [file MOL2-16-1857-s002.docx]

**Supplementary Figure Legends**

**
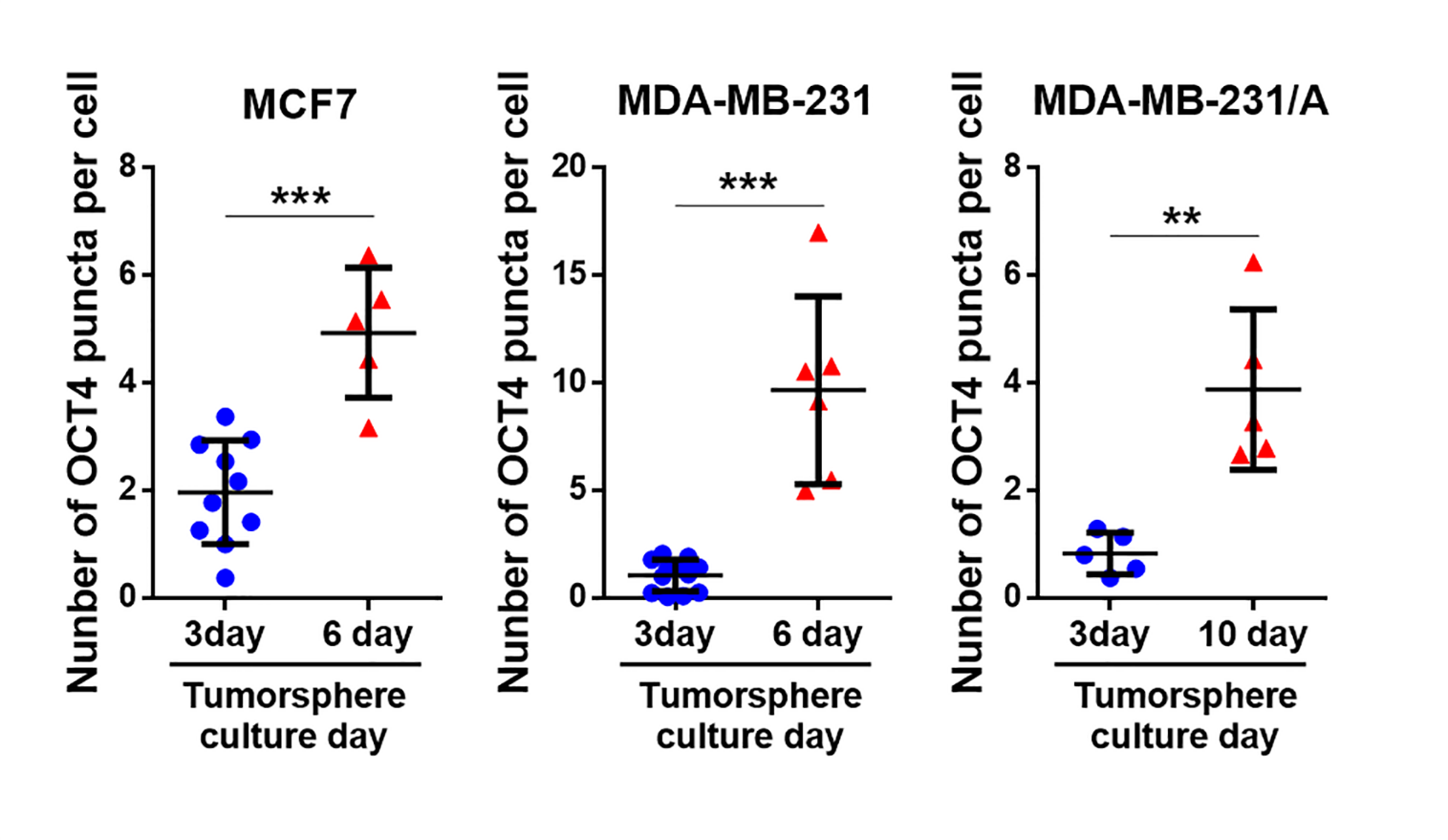
**

**Fig S1. The expression of OCT4 in breast cancer tumorspheres.**

The expression of OCT4 was quantified in tumorsphers of breast cancer cell lines. The number of OCT4 puncta was normalized with the number of DAPI-stained nuclei. Data were presented as mean ± SD. Statistical analyses were performed with one-tailed student’s t-test (***p* <0.01, ****p* <0.001).

**
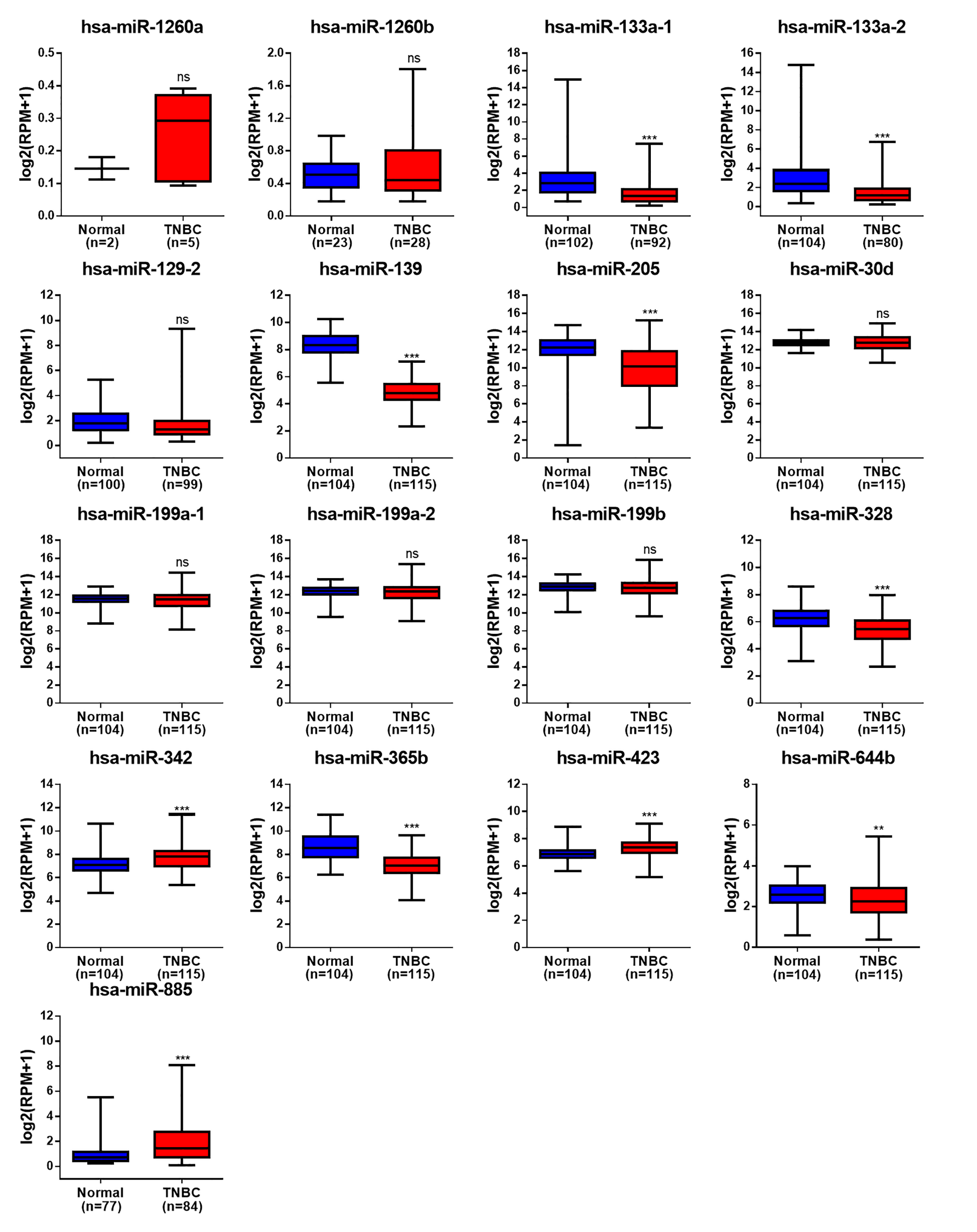
**

**Fig S2. The expression levels of miRNAs upregulated in MDA-MB-231 tumorspheres between normal and TNBC tissues.**

The TCGA analysis by MODBCdb showing the expression of 17 miRNAs, which are upregulated only in MDA-MB-231 tumorspheres. Statistical analyses were performed with one-tailed student’s t-test (***p* <0.01, ****p* <0.001; ns, non-significant difference).

**
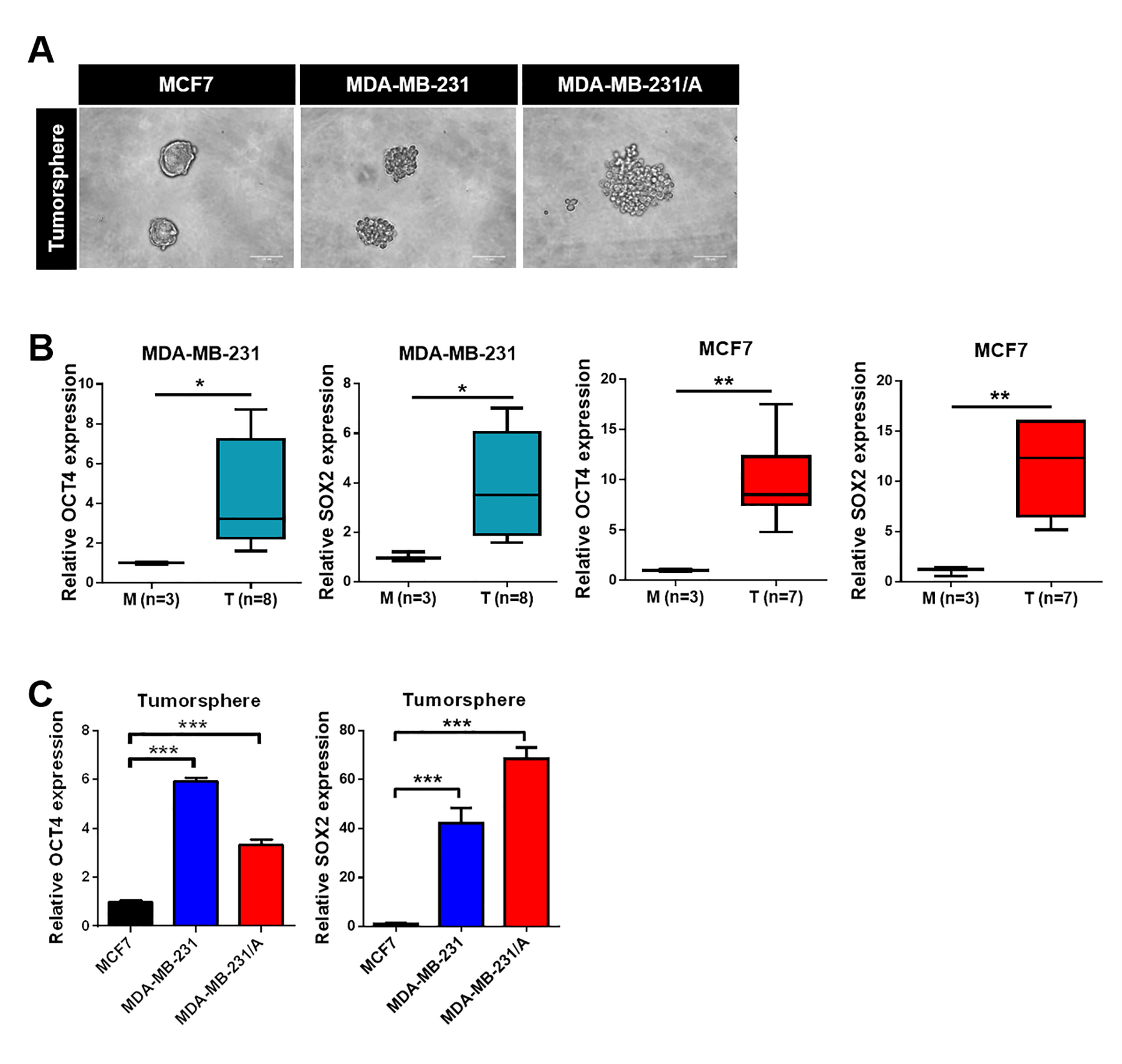
**

**Fig S3. The expression of cancer stem cell properties of breast cancer tumorspheres.**

(A) Representative images of breast cancer tumorspheres (magnification: ×20). Scale bar, 100 μm. (B) The mRNA levels of OCT4 and SOX2 in MDA-MB-231 and MCF7 cells were measured by qRT-PCR with specific primers. M, Monolayer; T, Tumorsphere. Statistical analyses were performed with one-tailed student’s t-test (**p <*0.05, ***p* <0.01). (C) mRNA levels of OCT4, and SOX2 in breast cancer tumorspheres. Data were presented as mean ± SD of three independent experiments. Statistical analyses were performed with one-tailed student’s t-test (****p* <0.001).

**
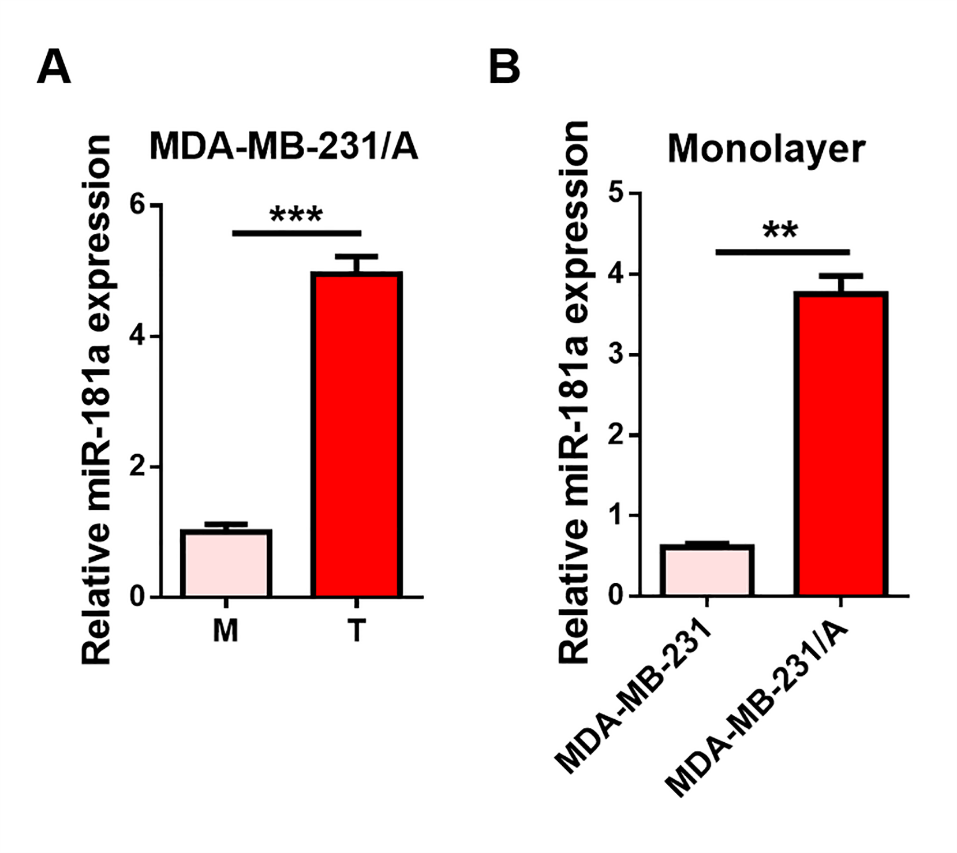
**

**Fig S4. miR-181a expression in MDA-MB-231 and MDA-MB-231/A cells.**

(A) miR-181a expression level in MDA-MB-231/A cells. M, Monolayer; T, Tumorsphere. Data were presented as mean ± SD of three independent experiments. Statistical analyses were performed with one-tailed student’s t-test (****p* <0.001). (B) The miR-181a expression levels in TNBC cell lines were measured using Taqman quantitative RT-PCR. Data were presented as mean ± SD of three independent experiments. Statistical analyses were performed with one-tailed student’s t-test (***p* <0.01).


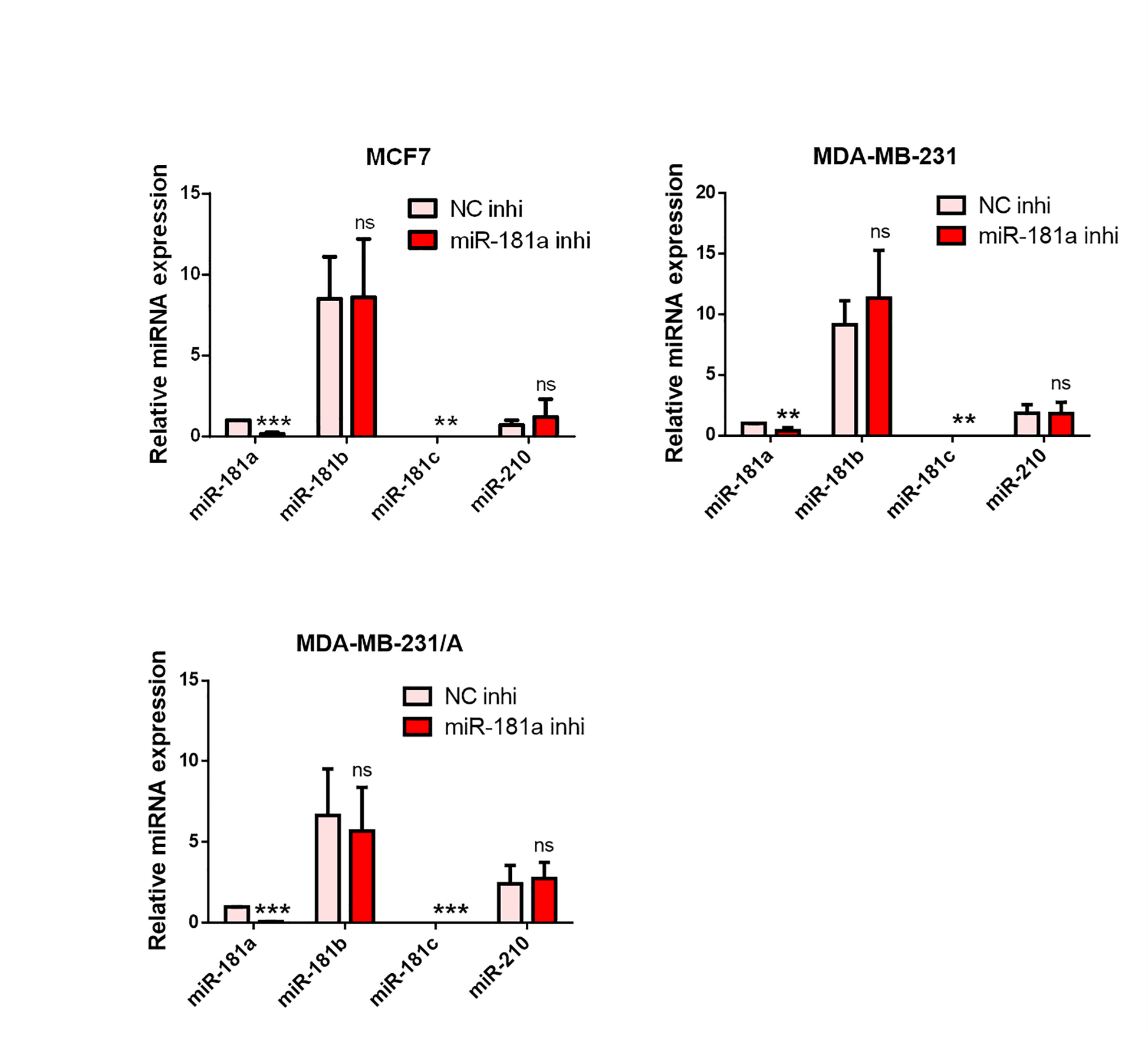


**Fig S5. The specificity of miR-181a inhibitor on breast cancer cells.**

The expression of other TNBC-upregulated miRNAs on breast cancer cell lines when miR-181a inhibitor was transfected. Data of MCF7 were presented as mean ± SD of four to five independent experiments. Data of MDA-MB-231 and MDA-MB-231/A were presented as mean ± SD of four independent experiments. Statistical analyses were performed with one-tailed student’s t-test (***p* <0.01, ****p* <0.001; ns, non-significant difference).

**
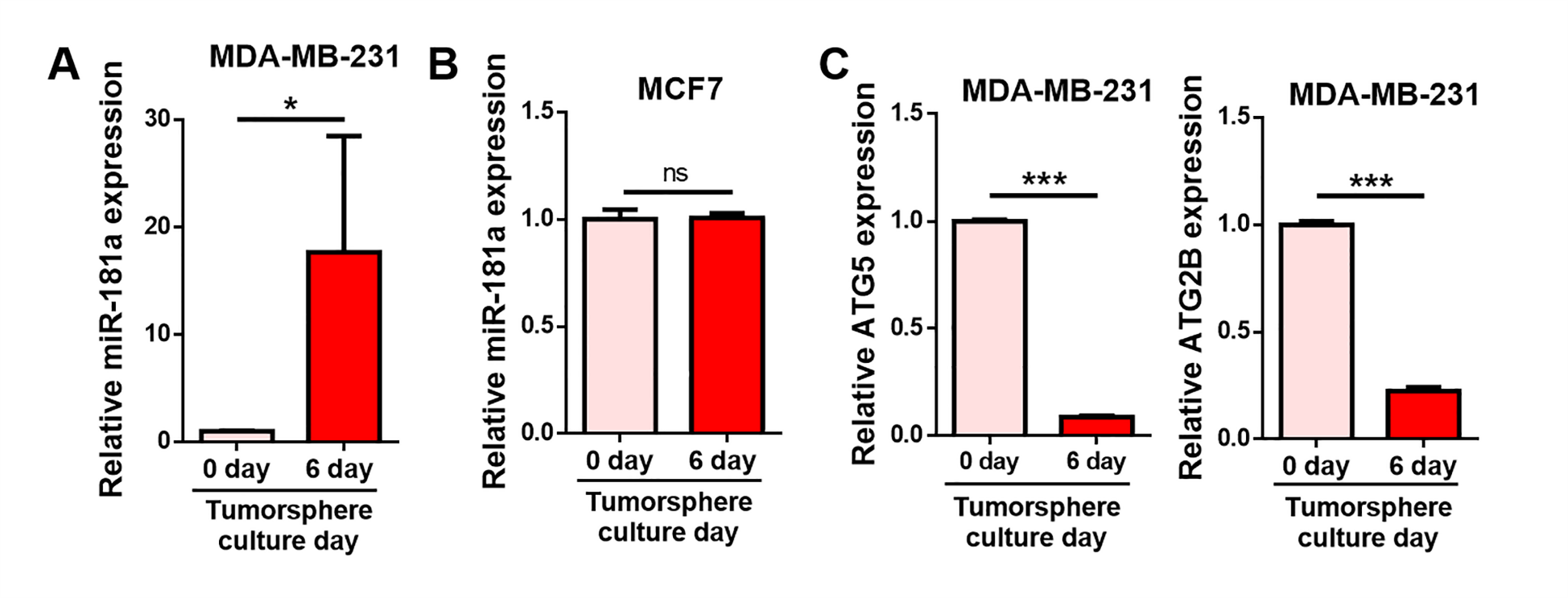
**

**Fig S6. The expression of miR-181a and autophagy target genes on in breast cancer tumorspheres.**

(A, B) Expression levels of miR-181a in MDA-MB-231 and MCF7 tumorspheres were assessed using Taqman qRT-PCR on days 0-6 of tumorsphere culture. Data were presented as mean ± SD of three independent experiments. Statistical analyses were performed with one-tailed student’s t-test (**p* <0.05; ns, non-significant difference). (C) ATG5 and ATG2G mRNA levels in MDA-MB-231 tumorspheres. Data were presented as mean ± SD of three independent experiments. Statistical analyses were performed with one-tailed student’s t-test (****p* <0.001).

**
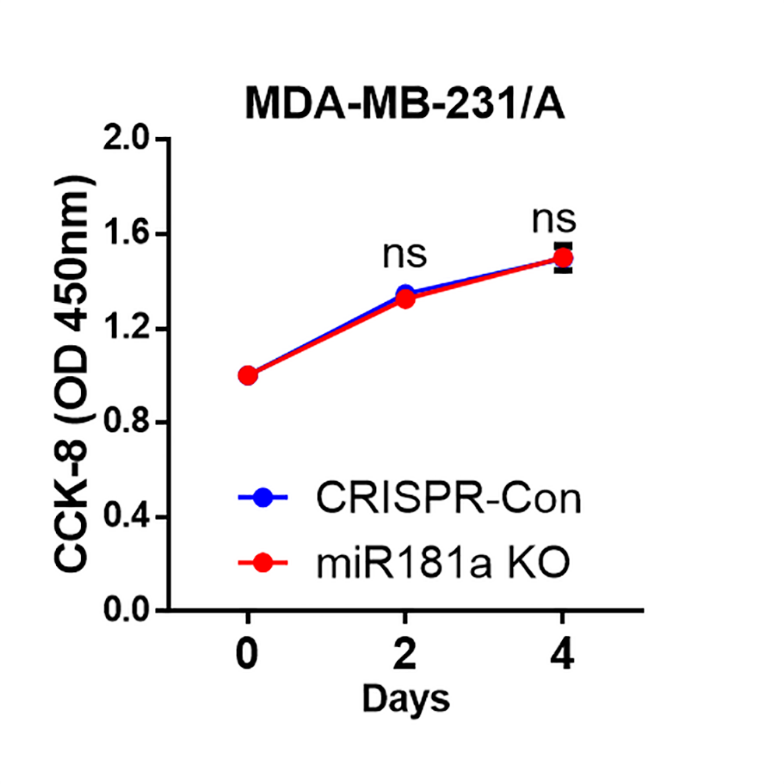
**

**Fig S7. The effect of miR-181a inhibition on the viability of MDA-MB-231/A cells.**

Cells were cultured in DMEM with 1% FBS, and the cell viability was measured using CCK-8 reagent. Data were presented as mean ± SD of four independent experiments. Statistical analyses were performed with one-tailed student’s t-test (ns, non-significant difference).


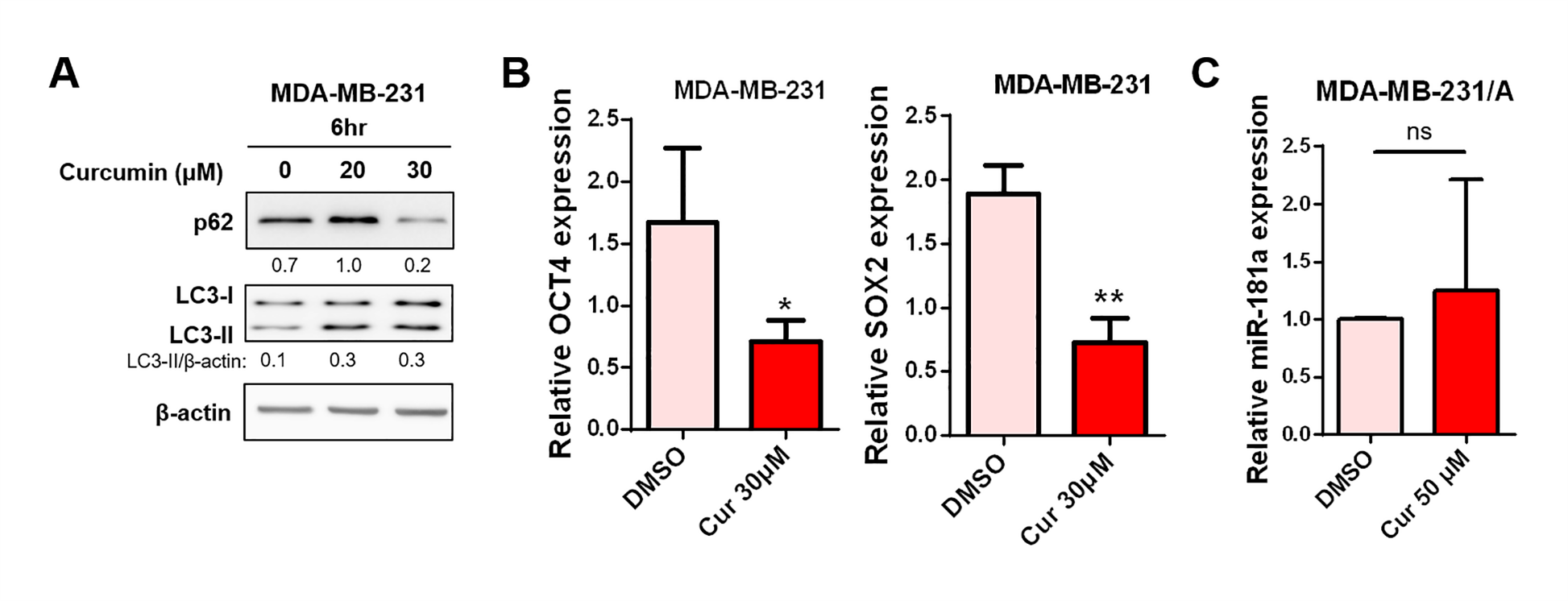


**Fig S8. The effects of curcumin on autophagy flux and cancer stemness of TNBC cells.**

(A) Treatment of MDA-MB-231 cells with different concentrations of curcumin. (B) mRNA expression levels of cancer stemness markers in curcumin-treated MDA-MB-231 cells. Data were presented as mean ± SD of three independent experiments. Statistical analyses were performed with one-tailed student’s t-test (**p* <0.05, ***p* <0.01). (C) miR-181a expression in curcumin-treated MDA-MB-231/A cells. Data were presented as mean ± SD of three independent experiments. Statistical analyses were performed with one-tailed student’s t-test (ns, non-significant difference).


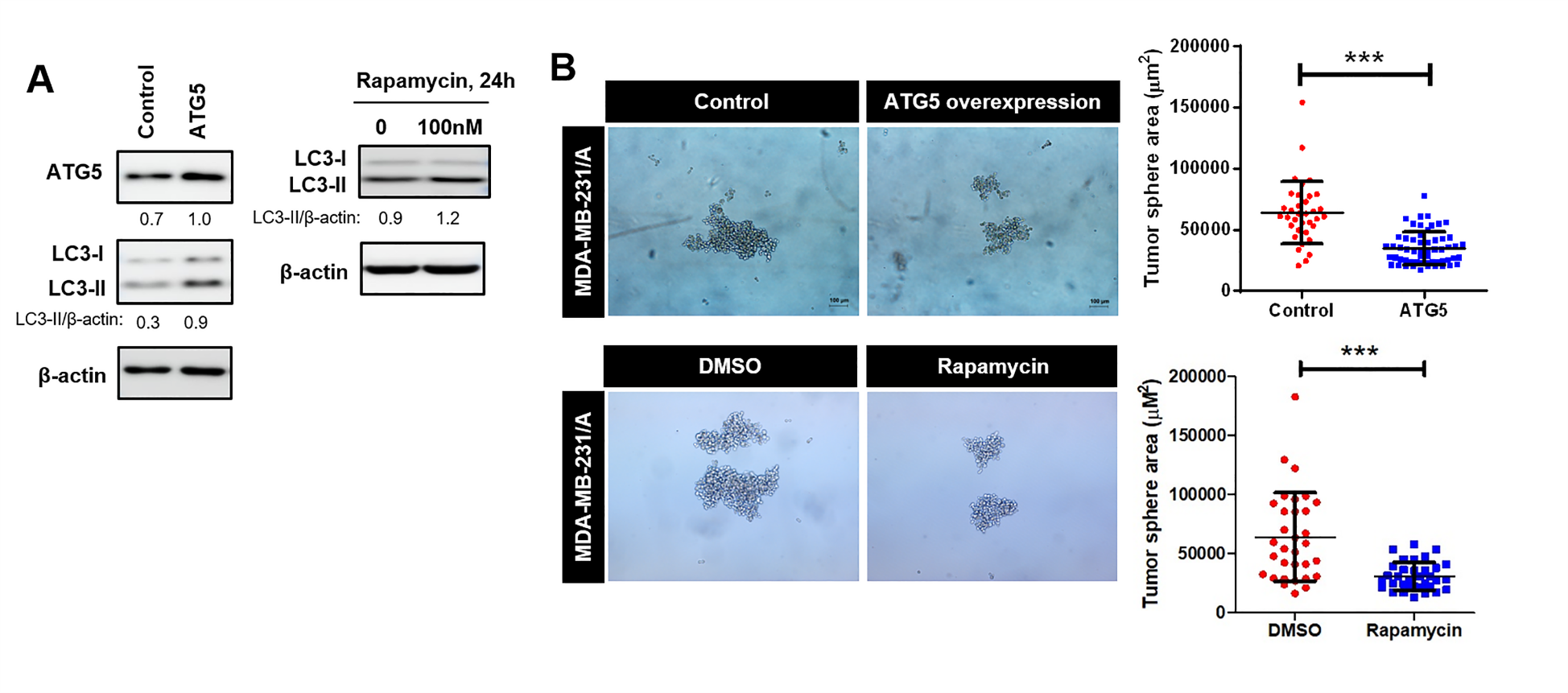


**Fig S9. The effects of ATG5 overexpression or rapamycin treatment on autophagy flux and turmorsphere of MDA-MB-231/A cells.**

(A) Protein levels of autophagy molecules in ATG5 overexpressed MDA-MB-231/A or rapamycin treated MDA-MB-231/A cells. (B) Representative images of MDA-MB-231/A tumorspheres transfected with ATG5 expression vector or treated with rapamycin. The sizes of tumospheres were quantified. Data were presented as mean ± SD. Statistical analyses were performed with one-tailed student’s t-test (****p* <0.001).


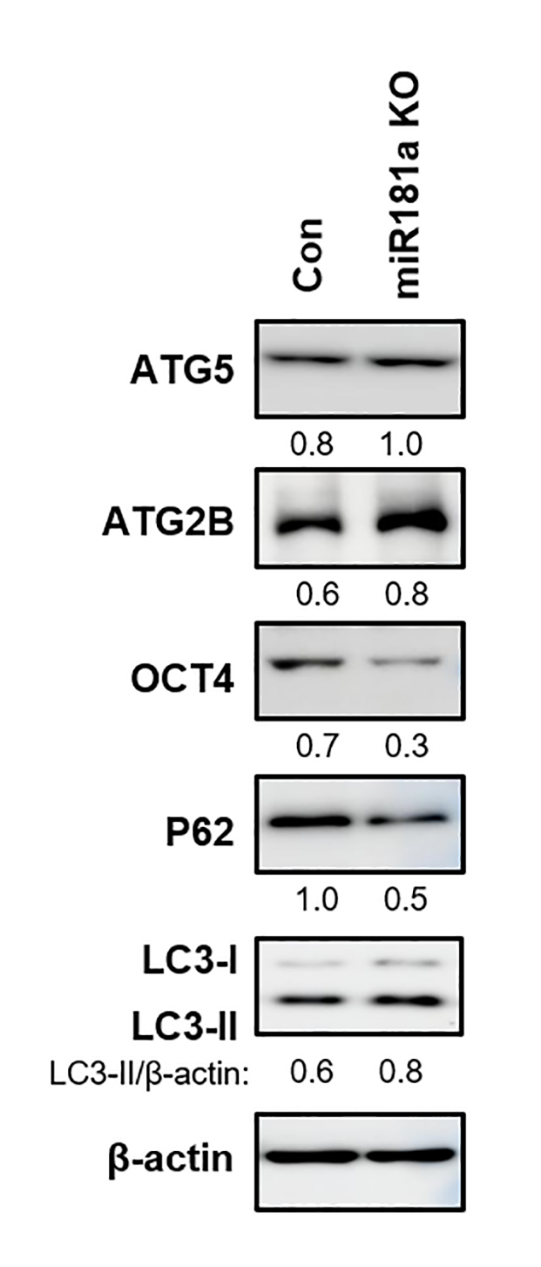


**Fig S10. Protein expression of autophagy and stemness marker in miR181a KO MDA-MB-231/A cells.**

Protein levels of ATG5, ATG2B, OCT4, p62 and LC3 in miR181a KO MDA-MB-231/A and control cells.

**Supplementary Tables**

| Name | Primer Sequences | | |
| --- | --- | --- | --- |
| Human 18s rRNA | **forward** | **5’-GTCGGCGTCCCCCAACTTCTT-3’** |  |
|  | **reverse** | **5’-CGTGCAGCCCCGGACATCTA-3’** |  |
| Human OCT4 | **forward** | **5’-CGTGCAGGCCCGAAAGAGA-3’** |  |
|  | **reverse** | **5’-GCTGGGCGATGTGGCTGAT-3’** |  |
| Human SOX2 | **forward** | **5’-CATGGGTTCGGTGGTCAAGTC-3’** |  |
|  | **reverse** | **5’-GGCGCCGGGGAGATACAT-3’** |  |
| Human NANOG | **forward** | **5’-TGCAAGAACTCTCCAACATCCTG-3’** |  |
|  | **reverse** | **5’-CTGCGTCACACCATTGCTATTCT-3’** |  |
| Human ATG5 | **forward** | **5’-TGGGCCATCAATCGGAAACTC-3’** |  |
|  | **reverse** | **5’-TGCAGCCACAGGACGAAACAG -3’** |  |
| Human ATG2B | **forward** | **5’-CCGTGGGCGAGGTTCTGC-3’** |  |
|  | **reverse** | **5’-CGTGGCGCCATTTCTGTGACT-3’** |  |

**Table S1. qRT-PCR primer sequences for each gene**

The sequences of qRT-PCR primers for 18s rRNA and the genes.

| Name | Primer Sequences | | |
| --- | --- | --- | --- |
| Human ATG5 WT-WT | **forward** | **5’-** **CTAGTTGTTTAAACGAGCTCTGTCCATATTGAATGTTGACCCA-3’** |  |
|  | **reverse** | **5’-** **CGACTCTAGACTCGAGCTGTACTGGCTATTCTTTTTGATT-3’** |  |
| Human ATG5 WT-MT | **forward** | **5’- CTAGTTGTTTAAACGAGCTCTGTCCATATTGAATGTTGACCCA-3’**  **5’-AAAAATGCCGATTTTTCTTATAAAAAATT-3’** |  |
|  | **reverse** | **5’-AATTTTTTATAAGAAAAATCGGCATTTTT-3’**  **5’-CGACTCTAGACTCGAGCTGTACTGGCTATTCTTTTTGATT-3’** |  |
| Human ATG5 MT-WT | **forward** | **5’-CTAGTTGTTTAAACGAGCTCTGTCCATATTACCTGTTGACCCA-3’** |  |
|  | **reverse** | **5’-CGACTCTAGACTCGAGCTGTACTGGCTATTCTTTTTGATT-3’** |  |
| Human ATG5 MT-MT | **forward** | **5’-CTAGTTGTTTAAACGAGCTCTGTCCATATTACCTGTTGACCCA-3’**  **5’-AAAAATGCCGATTTTTCTTATAAAAAATT-3’** |  |
|  | **reverse** | **5’-AATTTTTTATAAGAAAAATCGGCATTTTT-3’**  **5’-CGACTCTAGACTCGAGCTGTACTGGCTATTCTTTTTGATT-3’** |  |
| Human ATG2B WT | **forward** | **5’-** **AAACGAGCTCGCTAGCTTTGAGAAAGTGAATGTTGCAGAC-3’** |  |
|  | **reverse** | **5’-** **GCAGGTCGACTCTAGAAAAATATAAACAATTACATGGGCCC-3’** |  |
| Human ATG2B MT | **forward** | **5’-** **AAACGAGCTCGCTAGCTTTGAGAAAGTGCCGATTGC-3’** |  |
|  | **reverse** | **5’-** **GCAGGTCGACTCTAGAAAAATATAAACAATTACATGGGCCC-3’** |  |

**Table S2. The sequences of primers to amplify the 3’UTRs of the genes**

The primer sequences used in dual-luciferase assay for amplifying the wild-type (WT) 3’UTR and mutant-type (MT) 3’UTR.

| Name | Gene location | Sequences of oligos | |
| --- | --- | --- | --- |
| MIR181A1 | **Chromosome 1**  **NC_000001.11** | **Gene specific part of sgRNA** | **5’-GAGTAGAATTCTGAGTTTTGAGG-3’** |
|  |  | **Oligo 1** | **5’-CACCGGAGTAGAATTCTGAGTTTTG-3’** |
|  |  | **Oligo 2** | **5’-AAACCAAAACTCAGAATTCTACTCC-3’** |
| MIR181A2 | **Chromosome 9**  **NC_000009.12** | **Gene specific part of sgRNA** | **5’-TGTCGTCTGTAAGGACCCCAAGG-3’** |
|  |  | **Oligo 1** | **5’-CACCGTGTCGTCTGTAAGGACCCCA-3’** |
|  |  | **Oligo 2** | **5’-AAACTGGGGTCCTTACAGACGACAC-3’** |

**Table S3. The sequences of oligos for CRISPR-knockout of miR-181a**

The oligos ligated into lentiCRISPR v2 vector for constructing CRISPR-knockout (KO) cell line.

| Table S4. The list of predictive miR-181a target genes | | | | | |
| --- | --- | --- | --- | --- | --- |
| ARSB | BIRC6 | DRAM1 | ITGA3 | NRG1 | RB1 |
| ATG10 | BNIP3 | EIF2AK2 | ITGA6 | NRG3 | RPS6KB1 |
| ATG12 | CALCOCO2 | ERO1L | KIAA0226 | PARK2 | SERPINA1 |
| ATG16L1 | CANX | FAS | KLHL24 | PIK3C3 | SH3GLB1 |
| ATG2B | CASP1 | FKBP1A | LAMP2 | PRKAB1 | SIRT1 |
| ATG5 | CD46 | FOS | MAPK1 | PRKCD | TP63 |
| ATG7 | CDKN1B | GOPC | MAPK8 | PTEN | ULK1 |
| BAG1 | CHMP2B | GRID1 | MBTPS2 | PTK6 | ULK2 |
| BCL2 | DIRAS3 | HIF1A | MYC | RAB11A | ULK3 |
| BID | DLC1 | HSPA5 | NCKAP1 | RAB1A | UVRAG |
| BIRC5 | DNAJB1 | IFNG | NPC1 | RAB33B | WDFY3 |

**Table S4. Target prediction of miR-181a using TargetScan and HADb.**

66 of predictive targets of miR-181a, including ATG5 and ATG2B, were identified in HADb
