# Supplementary material for: Autophagy inhibits cancer stemness in triple‐negative breast cancer via miR‐181a‐mediated regulation of ATG5 and/or ATG2B
Source: Mol Oncol. 2022 Jan 26;16(9):1857–75. doi: 10.1002/1878-0261.13180 (PMC9067148; doi:10.1002/1878-0261.13180)
Supplement: Supplementary file 2 — Table S1. qRT‐PCR primer sequences for each gene. Table S2. The sequences of primers to amplify the 3’UTRs of the genes. Table S3. The sequences of oligos for CRISPR‐knockout of miR‐181a. Table S4. Target prediction of miR‐181a using TargetScan and HADb. [file MOL2-16-1857-s001.docx]

**Supplementary Tables**

| Name | Primer Sequences | | |
| --- | --- | --- | --- |
| Human 18s rRNA | **forward** | **5’-GTCGGCGTCCCCCAACTTCTT-3’** |  |
|  | **reverse** | **5’-CGTGCAGCCCCGGACATCTA-3’** |  |
| Human OCT4 | **forward** | **5’-CGTGCAGGCCCGAAAGAGA-3’** |  |
|  | **reverse** | **5’-GCTGGGCGATGTGGCTGAT-3’** |  |
| Human SOX2 | **forward** | **5’-CATGGGTTCGGTGGTCAAGTC-3’** |  |
|  | **reverse** | **5’-GGCGCCGGGGAGATACAT-3’** |  |
| Human NANOG | **forward** | **5’-TGCAAGAACTCTCCAACATCCTG-3’** |  |
|  | **reverse** | **5’-CTGCGTCACACCATTGCTATTCT-3’** |  |
| Human ATG5 | **forward** | **5’-TGGGCCATCAATCGGAAACTC-3’** |  |
|  | **reverse** | **5’-TGCAGCCACAGGACGAAACAG -3’** |  |
| Human ATG2B | **forward** | **5’-CCGTGGGCGAGGTTCTGC-3’** |  |
|  | **reverse** | **5’-CGTGGCGCCATTTCTGTGACT-3’** |  |

**Table S1. qRT-PCR primer sequences for each gene**

The sequences of qRT-PCR primers for 18s rRNA and the genes.

| Name | Primer Sequences | | |
| --- | --- | --- | --- |
| Human ATG5 WT-WT | **forward** | **5’-** **CTAGTTGTTTAAACGAGCTCTGTCCATATTGAATGTTGACCCA-3’** |  |
|  | **reverse** | **5’-** **CGACTCTAGACTCGAGCTGTACTGGCTATTCTTTTTGATT-3’** |  |
| Human ATG5 WT-MT | **forward** | **5’- CTAGTTGTTTAAACGAGCTCTGTCCATATTGAATGTTGACCCA-3’**  **5’-AAAAATGCCGATTTTTCTTATAAAAAATT-3’** |  |
|  | **reverse** | **5’-AATTTTTTATAAGAAAAATCGGCATTTTT-3’**  **5’-CGACTCTAGACTCGAGCTGTACTGGCTATTCTTTTTGATT-3’** |  |
| Human ATG5 MT-WT | **forward** | **5’-CTAGTTGTTTAAACGAGCTCTGTCCATATTACCTGTTGACCCA-3’** |  |
|  | **reverse** | **5’-CGACTCTAGACTCGAGCTGTACTGGCTATTCTTTTTGATT-3’** |  |
| Human ATG5 MT-MT | **forward** | **5’-CTAGTTGTTTAAACGAGCTCTGTCCATATTACCTGTTGACCCA-3’**  **5’-AAAAATGCCGATTTTTCTTATAAAAAATT-3’** |  |
|  | **reverse** | **5’-AATTTTTTATAAGAAAAATCGGCATTTTT-3’**  **5’-CGACTCTAGACTCGAGCTGTACTGGCTATTCTTTTTGATT-3’** |  |
| Human ATG2B WT | **forward** | **5’-** **AAACGAGCTCGCTAGCTTTGAGAAAGTGAATGTTGCAGAC-3’** |  |
|  | **reverse** | **5’-** **GCAGGTCGACTCTAGAAAAATATAAACAATTACATGGGCCC-3’** |  |
| Human ATG2B MT | **forward** | **5’-** **AAACGAGCTCGCTAGCTTTGAGAAAGTGCCGATTGC-3’** |  |
|  | **reverse** | **5’-** **GCAGGTCGACTCTAGAAAAATATAAACAATTACATGGGCCC-3’** |  |

**Table S2. The sequences of primers to amplify the 3’UTRs of the genes**

The primer sequences used in dual-luciferase assay for amplifying the wild-type (WT) 3’UTR and mutant-type (MT) 3’UTR.

| Name | Gene location | Sequences of oligos | |
| --- | --- | --- | --- |
| MIR181A1 | **Chromosome 1**  **NC_000001.11** | **Gene specific part of sgRNA** | **5’-GAGTAGAATTCTGAGTTTTGAGG-3’** |
|  |  | **Oligo 1** | **5’-CACCGGAGTAGAATTCTGAGTTTTG-3’** |
|  |  | **Oligo 2** | **5’-AAACCAAAACTCAGAATTCTACTCC-3’** |
| MIR181A2 | **Chromosome 9**  **NC_000009.12** | **Gene specific part of sgRNA** | **5’-TGTCGTCTGTAAGGACCCCAAGG-3’** |
|  |  | **Oligo 1** | **5’-CACCGTGTCGTCTGTAAGGACCCCA-3’** |
|  |  | **Oligo 2** | **5’-AAACTGGGGTCCTTACAGACGACAC-3’** |

**Table S3. The sequences of oligos for CRISPR-knockout of miR-181a**

The oligos ligated into lentiCRISPR v2 vector for constructing CRISPR-knockout (KO) cell line.

| Table S4. The list of predictive miR-181a target genes | | | | | |
| --- | --- | --- | --- | --- | --- |
| ARSB | BIRC6 | DRAM1 | ITGA3 | NRG1 | RB1 |
| ATG10 | BNIP3 | EIF2AK2 | ITGA6 | NRG3 | RPS6KB1 |
| ATG12 | CALCOCO2 | ERO1L | KIAA0226 | PARK2 | SERPINA1 |
| ATG16L1 | CANX | FAS | KLHL24 | PIK3C3 | SH3GLB1 |
| ATG2B | CASP1 | FKBP1A | LAMP2 | PRKAB1 | SIRT1 |
| ATG5 | CD46 | FOS | MAPK1 | PRKCD | TP63 |
| ATG7 | CDKN1B | GOPC | MAPK8 | PTEN | ULK1 |
| BAG1 | CHMP2B | GRID1 | MBTPS2 | PTK6 | ULK2 |
| BCL2 | DIRAS3 | HIF1A | MYC | RAB11A | ULK3 |
| BID | DLC1 | HSPA5 | NCKAP1 | RAB1A | UVRAG |
| BIRC5 | DNAJB1 | IFNG | NPC1 | RAB33B | WDFY3 |

**Table S4. Target prediction of miR-181a using TargetScan and HADb**

66 of predictive targets of miR-181a, including ATG5 and ATG2B, were identified in HADb.
